# A systematic review and meta‐analysis of prevalence and clinical features of upper gastrointestinal (UGI) tract Crohn's disease in adults compared to non‐UGI types

**DOI:** 10.1002/jgh3.12888

**Published:** 2023-05-10

**Authors:** Babak Tamizifar, Peyman Adibi, Maryam Hadipour, Vahid Mohamadi

**Affiliations:** ^1^ Isfahan Gastroenterology and Hepatology Research Center, Department of Internal Medicine Isfahan University of Medical Sciences Isfahan Iran; ^2^ Healthy Policy Research Center, Institute of Health Shiraz University of Medical Sciences Shiraz Iran

**Keywords:** Crohn's disease, prevalence, prognosis, systematic review, upper gastrointestinal tract

## Abstract

**Background and Aim:**

Crohn's disease is an inflammatory condition that affects the gastrointestinal (GI) system. This study aimed to determine the prevalence of upper gastrointestinal Crohn's disease (UGICD) and compare its features to non‐UGICD types.

**Methods:**

We conducted a systematic search in the databases PubMed, Web of Science, Scopus, and Google Scholar. The heterogeneity of prevalence estimates was examined, subgroup analyses were carried out, and meta‐analyses were conducted using random‐effects modeling. Prognostic data were qualitatively reviewed and combined.

**Results:**

Two‐thousand nine‐hundred and forty studies were retrieved and 32 studies were included in the final analysis. Pooled prevalence of UGICD was 15% (CI: 11–18%) among 14 509 patients. UGICD prevalence did not show any significant increase with time (*P* = 0.45). The most prevalent (38%, CI: 30–47%) behavior of UGICD was B1 (nonstricturing‐nonpenetrating), while the most common concurrent location was L3 (ileocolon) with a prevalence of 47% (CI: 34–59%). UGICD patients had higher stricturing phenotype (B2) compared to non‐UGICD (0.38 *vs* 0.30; *P* = 0.03). There was no significant difference in the prevalence of UGICD between patients classified according to the Montreal or Vienna classification. Stricturing phenotype was more common among Asian patients compared to Western patients (0.44 *vs* 0.24; *P* < 0.001). UGICD was a risk factor for surgery and drug therapy and was associated with an aggressive course of the disease and more resections. Pooled prevalence of UGICD was 15%.

**Conclusion:**

Nonstricturing‐nonpenetrating type was the most prevalent UGICD. UGICD patients had more complications and worse outcomes compared to non‐UGICD patients.

## Graphical abstract

This article is a systematic review and meta‐analysis of the prevalence and clinical features of upper gastrointestinal tract Crohn's disease (UGICD) in adults. The study aimed to determine the prevalence of UGICD and compare its features to Crohn's disease without UGI involvement.

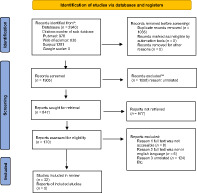

## Introduction

Crohn's disease is an inflammatory bowel disease that commonly affects the distal ileum and colon. However, the distribution of lesions can be in various parts of the gastrointestinal (GI) tract, from mouth to anus.^1^ Upper GI tract involvement is an infrequent disposition of the disease.^2^ In recent decades, because of the advances in diagnostic tools and imaging technologies, the upper gastrointestinal Chron's disease (UGICD) has been more recognized.^3^, ^4^ According to Zallot *et al*. UGICD has a worse outcome and is associated with more hospitalizations and surgeries.^5^ Several studies on the epidemiological characteristics of UGICD have been published in recent years.^6^, ^7^, ^8^

Although Chin *et al*.^9^ conducted a systematic review on the prevalence of UGICD, its risk factors, and outcomes, they failed to include almost all of the available documents. They searched for reports only in PubMed and Embase databases and ignored possible gray literature available and other less Westernized databases such as Scopus and Google Scholar. Accordingly, their study results may not be adequately robust. This systematic review aimed to determine the prevalence of UGICD and compare its features and outcomes with those of non‐UGICD.

## Methods

### Inclusion and exclusion criteria

A systematic review and meta‐analysis was designed and performed. We conducted a systematic search for available documents following the Preferred Reporting Items for Systematic Reviews and Meta‐Analyses (PRISMA) statement.

We searched for original documents on the prevalence, epidemiology, prognosis, clinical management, as well as its clinical features including location, behavior, and phenotype. Original studies with any observational design were included.

### Information source and search strategy

Based on the search technique used in the study, searches were conducted systematically. Scopus, PubMed, and Web of Science were searched online for published and indexed documents. To find online gray literature, Google Scholar was used. We also manually searched the list of references cited in the selected documents. All online searches were conducted on November 22, 2021. An online search strategy was designed using the following keywords: “upper gastrointestinal tract” AND “Crohn's disease”; “epidemiology”; “clinical management”; outcomes; and “clinical features.” The tools offered by each database were used to combine these keywords and their synonyms. The primary search strategy is described in the online Supplementary Material.

### Selection process

After retrieving the results of the searches and removing duplicate documents, the screening process was carried out independently by two authors according to prespecified criteria. If no consensus was available based on the document title and abstract, reviewers read the whole text, and in case of any disagreements about the inclusion of a document, the case was discussed by a methodologist.

### Risk‐of‐bias assessment

Risk‐of‐bias assessment was done by two authors independently. The NOS scale (Newcastle–Ottawa scale)^10^, ^11^ designed specifically for observational studies was used for critical appraisal. Epidemiologic features were assessed for possible bias through seven different questions, and any disagreements were discussed by a third author.

### Data items

Relevant data from the included documents were extracted into a piloted data form comprising the date of publication, first author name, geographical region (Western or Asian), country, period of the study, number of patients, family history of patients, female percentage, as well as disease location and phenotype according to Montreal^12^ or Vienna^13^ classification, concurrent location of upper gastrointestinal (L4) disease, diagnostic criteria for Crohn's disease, type of study, risk factors for UGICD, complications, and clinical or surgical management of the disease.

### Statistical methods

Pooled prevalence values for L1 (terminal ileum), L2 (colon), L3 (ileocolon), P (perianal), L4 (UGICD), and only L4 location (only UGI involvement) and the following B1 (nonstricturing‐nonpenetrating), B2 (stricturing), and B3 behavior (penetrating) were estimated by applying random‐effects modeling and illustrated in forest plots. Heterogeneity was assessed using the *I* ^2^ and Cochrane *Q* tests. Heterogeneity was defined as low ($I^{2}<25\%)$, moderate ($I^{2}:25–75\%)$, and significant ($I^{2}>75\%)$. Subgroup analyses were carried out considering the region, classification, and diagnostic methodology applying meta‐regression, and potential correlates of heterogeneity including female percent, smoker percent, and subgroup analyses were investigated. Forest plots were used to illustrate subgroup analysis based on region and involvement of the UGI tract (Supplementary Material—Forest plot). The Egger test was conducted and the funnel plots were used for the assessment of publication bias (Supplementary Material—Egger test and funnel plots). Data analyses were performed using Stata (StataCorp LP, College Station, TX, USA). Where meta‐analysis of the extracted data was not possible, qualitative synthesis was performed to summarize the information.

## Results

**Figure 1:**
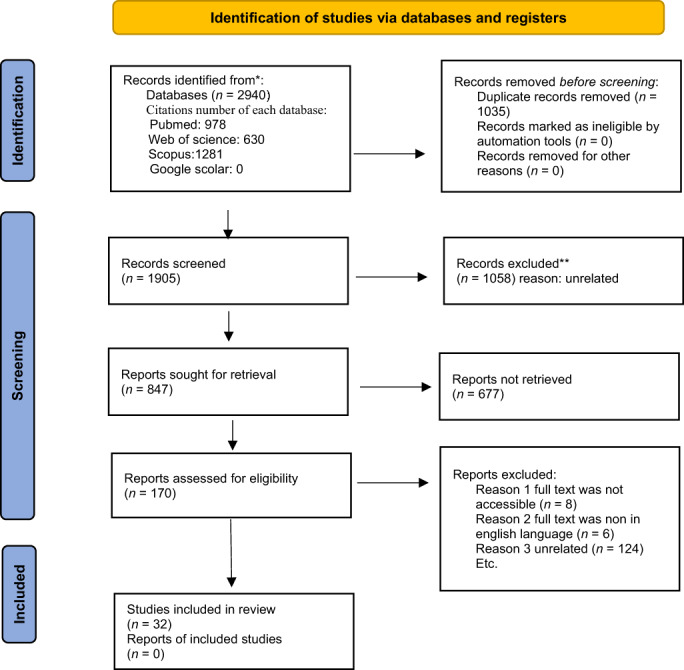
Flow diagram of the study according to the PRISMA method.

The flow diagram of the study was developed according to the PRISMA method^14^ (Fig. 1).

According to the flow diagram of the study, from the 2940 initial citations that were retrieved from the databases, 170 studies were selected for full‐text review and from those 170 papers, 32 essays with 35 Crohn's population were included in the final paper. The following three studies had two different Crohn's population bases: the Spekhorst study,^15^ the Jess study,^16^ and the Farkas study.^17^ Thirty studies were included in the meta‐analysis. Among the included papers, 21 (including 22 populations) studies were retrospective, 6 studies (including 8 populations) were prospective, and 1 study was cross‐sectional (Table 1). Eight papers were not accessible due to the access policies of the journal, so we emailed the authors for the full text, but the papers were not made available, thus they were excluded from the study.

**Table 1:** Summary of included studies

First author	Peri od	Number of cases	country	Region	Source of data	Study type	Classification	Diagnostic methodology	Quality score	L4*
Aljebreen *et al*.^18^	2014	497	Saudi Arabia	Asia	Data registry	Retrospective	Montreal	Clinical endoscopic radiology, histology	7\9	80
Chow *et al*.^19^	2009	132	China	Asia		Prospective	Montreal	Clinical endoscopic radiology, histology	8\9	30
Das *et al*.^20^	2008	141	India	Asia	Multi‐center hospital	Retrospective	Montreal	Clinical histology Endoscopic radiology	6\9	6
Dimitriu *et al*.^21^	2017	113	Romania	Western	Data registry		Montreal		6\9	14
Dorn *et al*.^22^	2004	535	USA	Western		Retrospective	Vienna	Clinical radiologic endoscopy, histology, laboratory	6\9	66
Gasche *et al*.^13^	2000	401	Norway and USA	Western	Data registry	Retrospective	Vienna		6\9	77
Moon *et al*.^23^	2015	1047	Korea	Asia	Data registry	Retrospective	Montreal	Clinical endoscopic radiology, histologic	8\9	95
Mokhtar *et al*.^24^	2019	132	Malaysia	Asia	data registry	retrospective	Montreal	Laboratory, radiology, endoscopy, histology	7\8	1
Leong *et al*.^25^	2004	80	China	Asia	Single medical center	Prospective	Vienna	Clinical endoscopy, histology, radiology	7\9	15
Farkas* *et al*.^17^	2016	100	China	Asia	Single medical center	Prospective	Montreal	Clinical radiology, endoscopy, histology, laboratory	7\8	4
Farkas* *et al*.^17^	2016	80	Hungary	Western	Single medical center	Prospective	Montreal	Clinical radiology, endoscopy, histology, laboratory	7\9	10
Pan *et al*.^26^	2020	869	China	Asia	Single medical center	Retrospective	Montreal	Clinical radiology, endoscopy, histology, laboratory tests, surgery report	8\9	519
Horjus Talabur Horje *et al*.^27^	2016	108	Netherlands	Western		Prospective	Montreal	Clinical radiology, endoscopy, histology,	8\9	44
Freeman *et al*.^28^	2001	877	Canada	Western	Single medical center	Retrospective	Vienna	Clinical radiology, endoscopy, histology	7\9	115
Lakatos L, et al.^29^	2011	163	Hungary	Western	multi‐center hospital	Prospective	Montreal	Clinical radiology, endoscopy, histology	8\9	4
Hilmi *et al*.^30^	2006	34	Malaysia	Asia	Sngle medical center	Retrospective	Vienna	Clinical radiology, endoscopy, histology, laboratory test	7\9	4
Sun *et al*.^31^	2019	246	China	Asia	Single medical center	Retrospective	Montreal	Clinical radiology, endoscopic histologic	7\9	80
Jess* *et al*.^16^	2007	52	Denmark	Western	Data registry	Retrospective	‐		6\9	10
Jess* *et al*.^16^	2007	209	Denmark	Western	Data registry	Retrospective	‐		6\9	17
Thia *et al*.^32^	2010	306	USA	Western	Data registry	Retrospective	Montreal	Clinical radiology, endoscopy, histology	7\9	13
Mao *et al*.^33^	2018	483	China	Asia	data registry	Retrospective	Montreal	Clinical radiology, endoscopy	8\9	185
Hwangbo *et al*.^34^	2010	81	Korea	Asia		Retrospective	Montreal	Clinical radiology, endoscopy, histology	6\9	15
Spekhorst* *et al*.^15^	2017	1825	CEU‐Netherlands	Western	Data registry	Prospective	Montreal		7\9	146
Spekhorst* *et al*.^15^	2017	143	NCEU‐Netherlands	Western	Data registry	Prospective	Montreal		7\9	23
Nóbrega *et al*.^35^	2018	141	Brazil		Single medical center	Retrospective	Montreal		7\9	5
Cao *et al*.^36^	2005	62	China	Asia	Single medical center	Retrospective	‐		6\9	11
de Barros *et al*.^37^	2017	179	Brazil	Brazil	Single medical center	Retrospective	Montreal		7\9	17
Louis *et al*.^38^	2001	297	Belgium	Western	Single medical center	Retrospective	Vienna		6\9	13
Freeman *et al*.^39^	2004	622	Canada	Western	Data registry	Retrospective	Vienna	Endoscopy, radiology histology, surgery reports	7\9	67
Greuter *et al*.^40^	2018	1638	Switzerland	Western	Data registry	Retrospective	Montreal		8\9	213
Lazarev *et al*.^41^	2013	2105	USA, Canada, Puerto Rico	Western	Data registry	Cross‐sectional	Montreal	Endoscopy, radiology, histology, surgery report	8\9	345
Sainz *et al*.^42^	2021	2562	Spain	Western	Data registry		Montreal	Endoscopy, histology, radiology	8\9	‐
Moon *et al*.^8^	2020	811	Korea	Asia	Single medical center	Retrospective	Montreal		8\9	24
Park^43^	2013	1403	Korea	Asia	Data registry	Retrospective	Montreal	Clinical endoscopy, radiology, histology	8\9	‐
Kim^44^	2018	1329	Korea	Asia	Multicenter hospital	retrospective	Montreal	Clinical endoscopy, radiology, histology	8\9	‐

The following studies marked with * had two different Crohn's population bases: Jess study,^16^ Farkas study,^17^ and Spekhorst.^15^ L4 marked by * represents the number of patients with upper GI involvement in each study.

We had two overall populations of Crohn's patients in our investigation. The first population comprised all the Crohn's patients who were studied in the included papers, and the second population (the L4 population) consisted of Crohn's patients with upper GI involvement.

In the whole population, the most common behavior was B1 (nonstricturing‐nonpenetrating) with a prevalence of 50% (CI: 41–58%). The most prevalent location of the disease was L3 (ileocolon) with 41% (CI: 36–45%) prevalence. UGICD prevalence was 15% (CI: 11–18%) (Table 2).

**Table 2:** Prevalence of Crohn's disease based on the location and behavior of the disease in the whole population

	No. of studies	Sample size	Heterogeneity index (*I* ^2^)	Pooled prevalence	95% CI	
Behavior						
B1 (non stricturing‐non penetrating)	29	16 635	99%	0.50	0.41	0.58
B2 (stricturing)	29	16 635	97%	0.24	0.20	0.29
B3 (penetrating)	29	16 635	98%	0.23	0.18	0.28
Location						
L1 (terminal ileum)	31	14 396	98%	0.25	0.20	0.31
L2 (colon)	31	14 396	96%	0.25	0.22	0.29
L3 (ileocolon)	31	14 396	96%	0.41	0.36	0.45
L4 (UGICD)	32	14 509	98%	0.15	0.11	0.18
P (perianal)	19	6707	89%	0.25	0.22	0.29

B1, nonstricturing‐nonpenetrating; B2, stricturing; B3, penetrating; L1, terminal ileum; L2, colon; L3, ileocolon; L4, upper GI; P, perianal.

In the L4 population, B1 (nonstructuring‐nonpenetrating) was the predominant behavior with 38% prevalence (CI: 30–47%). The most common concurrent location was L3 (ileocolon) with 47% (CI: 34–59%) (Table 3).

**Table 3:** Prevalence of Crohn's disease based on location and behavior of the disease in the L4 population

	No. of studies	Sample size	Heterogeneity index (*I* ^2^)	Pooled prevalence	95% CI	
Behavior						
B1 (non stricturing‐nonpenetrating)	10	1551	91%	0.38	0.30	0.47
B2 (stricturing)	10	1551	90%	0.33	0.25	0.41
B3 (penetrating)	10	1551	95%	0.25	0.16	0.33
Location						
L1 (concurrent terminal ileum)	14	1630	72%	0.25	0.20	0.30
L2 (concurrent colon)	14	1630	0%	0.12	0.11	0.14
L3 (concurrent ileocolon)	14	1630	96%	0.47	0.34	0.59
P (concurrent perianal)	5	1026	81%	0.26	0.19	0.33
Only L4 (only UGICD)	4	252	99%	0.29	0.00	0.72

B1, nonstricturing‐nonpenetrating; B2, stricturing; B3, penetrating; L1, terminal ileum; L2, colon; L3, ileocolon; Only L4, upper GI Crohn's disease without lower GI involvement; P, perianal.

### Comparison between the West and Asia

Fourteen studies including 16 populations (the Spephorst^15^ and Jess^16^ study had two populations) were from Western countries and 14 were from Asia. The pooled prevalence of UGICD in Asian patients was higher than in Western patients (0.18 [CI: 0.10–0.27] *vs* 0.12 [CI; 0.09–0.15]) though not significant (Supplementary Material—whole population data). The only significant difference between the pooled prevalence of measured parameters in Western and Asian patients with UGICD was the fact that the stricturing phenotype (B2) was more prevalent among Asian patients (0.44 *vs* 0.24; *P* < 0.001) (Table 4).

**Table 4:** Prevalence of characteristics of patients with upper GI Crohn's disease by region

Asia							Western							
	No. of studies	Sample size	Heterogeneity Index (*I* ^2^)	Pooled prevalence	95% CI			No. of studies	Sample size	Heterogeneity Index (*I* ^2^)	Pooled prevalence	95% CI		*P*‐value hetero geneity
Behavior							Behavior							
B1	5	838	92%	0.33	0.19	0.46	B1	5	714	91%	0.44	0.31	0.57	0.26
B2	5	838	62%	0.44	0.37	0.51	B2	5	714	86%	0.24	0.14	0.33	<0.001
B3	5	838	88%	0.17	0.09	0.25	B3	5	714	93%	0.3	0.16	0.43	0.11
Concurrent location							Concurrent location							
L1	6	842	13%	0.2	0.17	0.24	L1	6	128	62%	0.25	0.19	0.31	0.17
L2	6	842	0	0.12	0.1	0.14	L2	6	128	0	0.13	0.1	0.15	0.79
L3	6	842	96%	0.49	0.27	0.71	L3	6	128	90%	0.49	0.37	0.61	1
P	4	814	73%	0.23	0.17	0.3	P	‐	‐	‐	‐	‐	‐	
Only L4	1	95	‐	0.089	0.081	0.094	Only L4	3	157	‐	0.08	0.02	0.15	‐

B1, nonstricturing‐nonpenetrating; B2, stricturing; B3, penetrating; L1, terminal ileum; L2, colon; L3, ileocolon; Only L4, upper GI Crohn's disease without lower GI involvement; P, perianal.

### Comparison between Montreal and Vienna classification

Twenty studies including 22 populations (Spekhorst^15^ and Farkas^17^ studies had two papulations) were classified according to the Montreal classification and 7 studies were classified according to the Vienna classification. The pooled prevalence of UGI CD in studies with Montreal and Vienna classifications were respectively 0.16 (CI: 0.11–0.20) and 0.12 (CI: 0.09–0.16) (Supplementary Material—whole population data). There was no significant difference in the characteristics of UGICD patients, namely the prevalence of the disease, patient's age, phenotype, and concurrent location of the disease, between studies classified according to Montreal and Vienna classifications (Table 5).

**Table 5:** Prevalence of characteristics of patients with upper GI Crohn's disease by classification

Montreal							Vienna							
	N. of studies	Sample size	Heterogeneity Index (I^2^)	Pooled Prevalence	95% CI			N. of studies	Sample size	Heterogeneity Index (I^2^)	Pooled Prevalence	95% CI		*P*‐value hetero geneity
Behavior							Behavior							
B1	8	1409	91%	0.41	0.31	0.5	B1	‐	‐	‐	‐	‐	‐	‐
B2	8	1409	87%	0.36	0.28	0.44	B2	‐	‐	‐	‐	‐	‐	‐
B3	8	1409	92%	0.18	0.1	0.25	B3	‐	‐	‐	‐	‐	‐	‐
Concurrent location							Concurrent location							
L1	10	1430	80%	0.27	0.21	0.33	L1	3	186	0	0.19	0.14	0.25	0.07
L2	10	1430	0	0.13	0.11	0.14	L2	3	186	0	0.11	0.06	0.15	0.49
L3	10	1430	96%	0.42	0.28	0.57	L3	3	186	0	0.63	0.56	0.7	0.01
P	5	1027	81%	0.26	0.19	0.33	P	‐	‐	‐	‐	‐	‐	
Only L4	1	95	‐	0.089	0.081	0.094	Only L4	2	143	‐	0.11	0.06	0.16	‐

B1, nonstricturing‐nonpenetrating; B2, stricturing; B3, penetrating; L1, terminal ileum; L2, colon; L3, ileocolon; Only L4, upper GI Crohn's disease without lower GI involvement; P, perianal.

### Comparison between L4 and non‐L4 populations

The L4 population comprised Crohn's patients with upper GI involvement and the non‐L4 population consisted of Crohn's patients without upper GI involvement. The stricturing phenotype (B2) was significantly more common in the L4 population compared to the non‐L4 population (0.38 *vs* 0.30; *P* = 0.03); also, isolated colon involvement (L2) was significantly higher among the non‐L4 papulation (0.25 *vs* 0.13; *P* = 0.01) (Table 6).

**Table 6:** Comparison between characteristics of Crohn's patients with upper GI and non‐upper GI involvement

Patients with upper GI involvement							Patients without upper GI involvement							
	No. of studies	Sample size	Heterogeneity index (*I* ^2^)	Prevalence	95% CI			No. of studies	Sample size	Heterogeneity Index (*I* ^2^)	Prevalence	95% CI		*P*‐value hetero geneity
Behavior							Behavior							
B1	8	2252	94%	0.4	0.3	0.49	B1	8	6594	99%	0.48	0.28	0.67	0.45
B2	8	2252	87%	0.38	0.32	0.45	B2	8	6594	92%	0.3	0.26	0.34	0.03
B3	8	2252	92%	0.17	0.11	0.23	B3	8	6594	99%	0.21	0.11	0.31	0.49
Location							Location							
L1	7	2172	69%	0.23	0.19	0.28	L1	7	4886	99%	0.23	0.11	0.34	0.92
L2	7	2172	0%	0.13	0.11	0.15	L2	7	4886	98%	0.25	0.17	0.33	0.01
L3	7	2172	98%	0.5	0.33	0.67	L3	7	4886	76%	0.51	0.48	0.54	0.92
P	5	1028	82%	0.26	0.19	0.33	P	5	2340	84%	0.31	0.26	0.37	0.27

B1, nonstricturing‐nonpenetrating; B2, stricturing; B3, penetrating; L1, terminal ileum; L2, colon; L3, ileocolon; P, perianal.

### L4 trend

There was no significant relationship between L4 pooled prevalence and time (*P* = 0.45).

### Meta‐regression

#### L1

Although L1 prevalence did not differ significantly between L4 and non‐L4 in simple subgroup analysis, according to data in meta‐regression, all the three variables, namely female percent, smoker percent, and subgroup (analysis between L4 and non‐L4 group), significantly affect the heterogeneity (differences) of L1 prevalence between L4 and non‐L4 population (subgroup *P* = 0.02; smoker percent *P* = 0.01; female percent *P*  <0.001).

#### L2

The effect of the subgroup was not significant on the heterogeneity of L2 prevalence (*P* = 0.45) but the smoker percent (*P* = 0.02) and female percent (*P* < 0.001) were both significantly correlated with the heterogeneity of measured L2 prevalence. (In subgroup analysis, the subgroup effect was significant, but when these three variables were analyzed together, the significance of the subgroup effect disappeared).

#### L3

When the variables smoker percent, female percent, and subgroup were analyzed simultaneously, all three of them (smoker *P* < 0.001, female percent *P* < 0.001, subgroup *P* < 0.001) were significantly effective on the heterogeneity of L3 prevalence between L4 and non‐L4 population.

#### B1

Also, all the mentioned variables were significantly effective in the heterogeneity of B1 prevalence (smoker percent *P* = 0.00; female percent *P* < 0.001; subgroup *P* = 0.05).

#### B2

In B2, the only effective variable on the heterogeneity of prevalence was female percent (female percent *P* < 0.001, subgroup *P* = 0. 93, smoker *P* = 0.59).

#### B3

Subgroup was the only variable significantly responsible for making a difference in the heterogeneity of B3 prevalence between the L4 and non‐L4 populations. (In simple subgroup analysis, the subgroup was not effective in the heterogeneity of B3 prevalence, but after controlling the confounder variables, the subgroup effect became significant: subgroup prevalence = 0.05; female prevalence = 0.72; smoking prevalence = 0.35.)

#### P

In meta‐regression, none of the variables, namely female percent, smoker percent, and subgroup, was correlated with the heterogeneity of prevalence between L4 and non‐L4 (see Table 7).

**Table 7:** Meta‐regression

Parameters	Excluded effect	*P*‐value
L1	Subgroup	0.02
	Female %	0.00
	Smoker %	0.01
L2	Subgroup	0.45
	Female %	0.00
	Smoker %	0.02
L3	Subgroup	0.00
	Female %	0.00
	Smoker %	0.00
B1	Subgroup	0.05
	Female %	0.00
	Smoker %	0.00
B2	Subgroup	0.93
	Female %	0.00
	Smoker %	0.59
B3	Subgroup	0.05
	Female %	0.72
	Smoker %	0.35
P	Subgroup	0.91
	Female %	0.14
	Smoker %	0.37

In subgroup analysis, comparisons between L4 and non‐L4 populations based on the mentioned variables in Table 7 were carried out. The L4 population was defined as Crohn's patients with upper GI involvement and non‐L4 was defined as Crohn's patients without upper GI involvement.

### Outcome

All the relevant qualitative data on UGICD outcome and its comparison to non‐UGICD are given in Table 8.

**Table 8:** Comparison between the outcome of UGICD and non‐UGICD population

Authors	Period	Comparison between UGICD and non‐UGICD
Louis *et al*.^38^	2010	Proximal small bowel and upper gastrointestinal tract locations are associated with the risk of recurrence and surgery.
Chow *et al*.^19^	2009	The L4 phenotype independently predicted further hospitalization (adjusted hazards ratio [HR]: 2.1; 95% CI: 1.3–3.5). The cumulative probability of major surgery was significantly higher in the L4 than in the non‐L4 group (*P* = 0.0001)
Greuter *et al*.^40^	2018	GI tract involvement showed a disease course similar to control patients (hazard ratio [HR] for any complications 0.887, 95% confidence interval [CI] 0.409–1.920)
Lazarev *et al*.^41^	2013	The jejunal disease is a significantly greater risk factor for stricturing disease and multiple abdominal surgeries than either EGD or ileal (without proximal) disease. Jejunal site (stricturing risks [OR 2.90; 1.89–4.45], multiple surgeries risk [OR 2.39; 1.36–4.20])
Barros *et al*.^37^	2017	L4 is a relative risk (RR) for stricturing behavior 2.11 (1.18–3.76) (*P*‐value = 0.001) and complicating disease 1.93 (1.29–2.88) (*P*‐value <0.012)
Sun *et al*.^31^	2019	L4 disease was an independent risk factor for abdominal surgery within 1 year post diagnosis (OR 6.335; 95% CI 3.862–10.390) and was associated with higher rates of abdominal surgery (41.3% *vs* 11.4%, *P* < 0.001) but similar rates of hospitalization within 1 year post‐diagnosis compared to non‐L4 disease. Jejunal and proximal ileal involvement was associated with stricturing behavior (*P* = 0.034, *P* < 0.001) and a higher abdominal surgery rate (both *P* < 0.001). Early outcomes are worse for L4 than for non‐L4 disease thus more aggressive initial therapy is needed to improve L4‐disease prognosis.
Mao *et al*.^33^	2018	Compared to non‐L4 patients, L4 patients were more likely to have intestinal surgeries during follow‐up (31% *vs* 16%, *P* < 0.001). L4‐jejunal (HR 3.08; 95% CI 1.30–7.31) and L4‐proximal ileal disease (HR 1.83; 95% CI 1.07–3.15) were independent predictors for intestinal resection in multi‐variable analysis.
Kim *et al*.^44^	2018	compared to patients without proximal small bowel involvement, those with small bowel involvement: Were more likely to display stricturing behavior (19.8% *vs* 12.7%, *P* = 0.020). Had more upper gastrointestinal involvement (OR, 1.643; 95% CI: 1.008–2.677) and lower surgery‐free survival (10‐year surgery‐free survival: 58.4% *vs* 67.7%, respectively, *P* < 0.001)
Park^43^	2013	Jejunal group in comparison to non‐jejunal group: Had more ileal location (28.3 *vs* 20.6%, *P* < 0.001) and stricturing behavior (16.7 *vs* 9.4%, *P* = 0.001). In univariate analyses, had significantly higher cumulative probabilities of treatment with corticosteroids (*P* = 0.014) and thiopurines (*P* = 0.008), the first major surgery (*P* = 0.021), and the first hospitalization (*P* = 0.015) In multivariate analyses, had greater use of corticosteroids (hazard ratio, 1.24; 95% CI: 1.02–1.50) and thiopurines (hazard ratio, 1.26; 95% CI, 1.06–1.49), higher incidence rates of strictureplasties (relative risk [RR], 2.52; 95% CI, 1.60–3.96) and hospitalizations (RR, 1.29; 95% CI, 1.14–1.47), and longer hospitalization duration (RR, 1.30; 95% CI, 1.25–1.34)

### Risk factors

All the relevant qualitative data on UGICD risk factors are given in Table 9.

**Table 9:** Risk factors for developing UGI_CD

Authors	period	Odds ratio for UGICD development
Pan *et al*.^26^	2020	According to logistic regression, body weight (OR 1.054), disease course (OR 1.010), stricturing behavior (OR 4.998), and tomato intolerance (OR 1.313) were the independent risk factors for L4 involvement. L4 disease mainly affects males and those with prolonged disease course, stricturing behavior, higher weight, BMI, albumin levels, and food intolerance (FI).
Greuter *et al*.^40^	2018	In a multivariate logistic regression model, male sex and diagnosis between 2009 and 2016 *versus* diagnosis between 1955 and 1995 period were independent predictors for the presence of upper GI tract involvement at CD diagnosis (odds ratio [OR] 1.600, *P* = 0.021 and [OR] 2.686, *P* < 0.001, respectively), whereas adult age, was a negative predictor (OR 0.388, *P* = 0.001).

## Discussion

This study is a systematic review and meta‐analysis conducted on UGICD prevalence and its risk factors and outcome. Thirty studies comprising 33 Crohn's populations with 17 071 patients were included in the meta‐analysis. The pooled prevalence of UGICD was 15% (CI: 11–18%) in 14 509 patients across 32 populations, consistent with a 13% pooled prevalence of UGICD in the Chin and colleagues′ study,^9^ which is a previous systematic review conducted on this subject in 2021.

Although the L4 (upper GI involvement) prevalence trend did not show any significant evidence of increase with time (*P* = 0.45), studies after 2019 had a nonsignificantly higher L4 prevalence (15% in studies between 2015 and 2019 *vs* 24% in studies after 2019) and also pooled prevalence of UGICD based on the Montreal classification; the newer classification was nonsignificantly higher (*P* = 0.32) (16 *vs* 12%) in contrast to the Vienna classification. This late increase in L4 prevalence can be due to the limitations of our review process. Another hypothesis is that recent advances in diagnostic and imaging technologies are leading to more accurate detection of UGI involvement in Crohn's patients; however, to the best of our knowledge, there is no convincing study on the changes in UGICD prevalence rate in recent years, and our findings need to be verified in future studies. There was no significant difference in measured parameters such as age, phenotype, and location of the disease between UGICD patients in studies classified according to the Montreal or Vienna classification.

The pooled prevalence of UGICD in Asian and Western countries did not have any significant difference; however, the prevalence of upper GI involvement in the Asian population was higher (18 *vs* 12%; *P* = 0.17). Similar findings regarding the higher prevalence of UGICD in Asia have also been reported in Chin and colleagues' review^9^ and they are in accordance with the rapid increase in the incidence and prevalence of inflammatory bowel disease (IBD) in Asian countries over the recent decades.^45^, ^46^

The only significant difference between the pooled prevalence of measured parameters between Western and Asian patients with UGICD was the fact that stricturing phenotype (B2) was more common among Asian patients (0.44 *vs* 0.24; *P* < 0.001). This finding is congruent with Ng and colleague's large‐scale population‐based study, which found that complicated CD (stricturing, penetrating, or perianal disease) was more common in Asia than Australia (52 *vs* 24%; *P* = 0.001). According to their result, IBD can be as severe or more severe in Asia than in the West.^47^

Based on the results, UGICD patients compared to non‐UGICD patients had more stricturing behavior (B1) (0.38 *vs* 0.30; *P* = 0.03), which is in agreement with our overall prognostic data in which UGI involvement in Crohn's patients is a risk factor for more surgeries and resection therapies.^31^, ^37^, ^41^ The pooled prevalence of isolated colon involvement (L2) was significantly higher among patients without UGI involvement (0.25 *vs* 0.13; *P* = 0.01). This could be due to the presence of the more extensive ileocolonic form of the disease with small bowel involvement in UGICD, as suggested in the Park and Kim study, which was included our studies in the qualitative data.^43^, ^44^

Male sex was a common risk factor in the two included studies on the risk factors for the occurrence of UGI involvement,^26^, ^40^ Yet, since there is not adequate data on the subject, the interpretation is inconclusive.

According to the result of meta‐regression, based on the female percent, smoker percent, and L4 and non‐L4 subgroup analysis, female percent was significantly effective in the heterogeneity of pooled prevalence of L1, L2, L3, B1, and B2 between the two population of L4 and non‐L4. The smoker percent and the subgroup analysis significantly affect the heterogeneity of pooled prevalence of L1, L2, L3, B1, and L1, L3, respectively. To the best of our knowledge, there is no data available in this regard and thus our findings need to be verified in future studies.

In the qualitative segment of the study (Tables 8 and 9), the studies of Kim *et al*.^44^ Lazarev *et al*.^41^ and Park *et al*.^43^ compared Crohn's disease with and without small bowel involvement. Lazarev and colleagues^41^ in a cross‐sectional study of the IBD genetic consortium compared jejunal with non‐jejunal Crohn's disease, and according to their results, jejunal involvement is a significantly greater risk factor for stricturing behavior and multiple abdominal surgeries. Park and coworkers^43^ in a retrospective study in Korea compared the prognosis of two groups of jejunal and non‐jejunal Crohn's patients. According to their report, jejunal patients had more ileal location and stricturing behavior than the non‐jejunal patients. In their study, on both univariate and multivariate analysis, jejunal involvement was independently associated with more corticosteroid and thiopurine use, more surgery, and more hospitalizations. Kim and their colleagues^44^ in the connect cohort study compared Crohn's patients with and without proximal small bowel involvement. According to their results, patients with small bowel involvement had poorer surgery‐free survival and more upper GI involvement. All three mentioned studies maintain the fact that the involvement of small bowel disease, and particularly jejunum, had a poor prognosis and worse outcome in contrast to Crohn's patients without small bowel involvement.

Regarding the correspondence of clinical features including prognosis and outcome between UGICD and non‐UGICD, the studies of Louis *et al*.^38^ Chow *et al*.^19^ Mao *et al*.^33^ and Sun *et al*.^31^ demonstrate that UGICD in contrast to non‐UGICD was associated with significantly more surgeries; besides, the studies of de Barros *et al*.^37^ Lazarev *et al*.^41^ and Sun *et al*.^31^ suggest that UGICD patients had more stricturing behavior in contrast to non‐UGICD patients. Furthermore, studies of Chow *et al*.^19^ and Park *et al*.^43^ found that UGICD patients had a longer duration of hospitalization than non‐UGICD patients. However, according to Sun *et al*.^31^ and Greteur *et al*.^40^ there were no significant differences between UGICD and non‐UGICD disease courses. According to Sun and colleagues,^31^ UGICD patients had similar rates of hospitalization compared to non‐UGICD patients, and according to the Greteur *et al*.^40^ study, PCD (proximal Crohn's disease) group had a disease course similar to that of the non‐PCD group of patients. However, the findings of these last two works are in contrast with those of the rest of the included studies in the review; nevertheless, seven of nine papers included in our study maintain that UGICD was associated with more surgeries, complications, and worse prognosis. Thus the overall result of the included studies favor the conclusion that UGICD is a risk factor for surgery, resection, and drug therapy and has a more aggressive disease course.

In contrast to Chin's study, this paper has conducted the first review in three databases, namely the Web of Science, Scopus, and Google Scholar, and included six (23%) more reports with nine (34%) more population bases of UGICD, more comprehensive literature search, and a convincing number of eligible reports, thus increasing the validity of our results and making it more principled; however, the overall findings of our study are supportive of their results. Besides that, this review has three distinct populations of Crohn's patients with their measured demographic characteristics according to Montreal and Vienna classifications: The first and the main population is the L4 or UGICD population, and the second and the last populations are the non‐L4 and the whole populations, respectively. We have carried out two comparisons: comparison of demographic characteristics between the L4 and non‐L4 populations, and a meta‐regression analysis between the two populations of L4 and non‐L4 based on the variables female percent, smoking, and subgroup analysis; however, due to the originality of this data, the conclusions of this section need to be verified in future original studies. And our qualitative findings in Tables 8 and 9 regarding the outcome and risk factor of UGICD patients due to limitations of the qualitative review process have to be appraised more in future studies.

## Conclusions

In light of the 15% prevalence of UGICD according to our analysis, clinicians should be aware of possible upper GI involvement and conduct required investigations in the early phase of the disease to prevent complications, if necessary.

## Ethics approval

Committee of Medical Ethics, Isfahan Medical Institute, approved our study. Our ethical code is IR.ARI.MUI.REC.1400.095.

## Supporting information


**Data S1.** Supplementary file

**Data S2.** Supplementary file

## Data Availability

Data and materials of our cases are available in Supplementary Material.
