# Supplementary material for: A systematic review and meta‐analysis of prevalence and clinical features of upper gastrointestinal (UGI) tract Crohn's disease in adults compared to non‐UGI types
Source: JGH Open. 2023 May 10;7(5):325–36. doi: 10.1002/jgh3.12888 (PMC10230113; doi:10.1002/jgh3.12888)

**All Forest Plots and Funnel Plots for prevalence Based on the whole population:**

Prevalence of L1


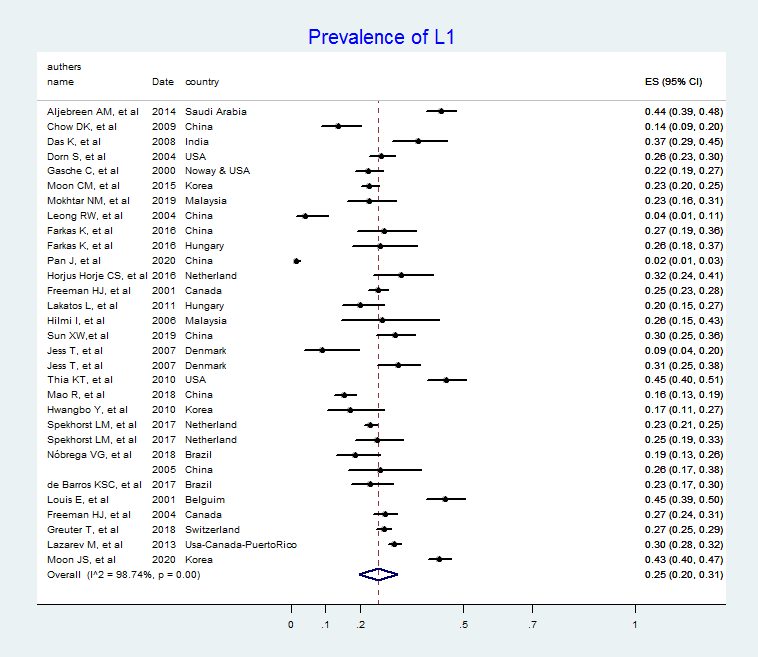


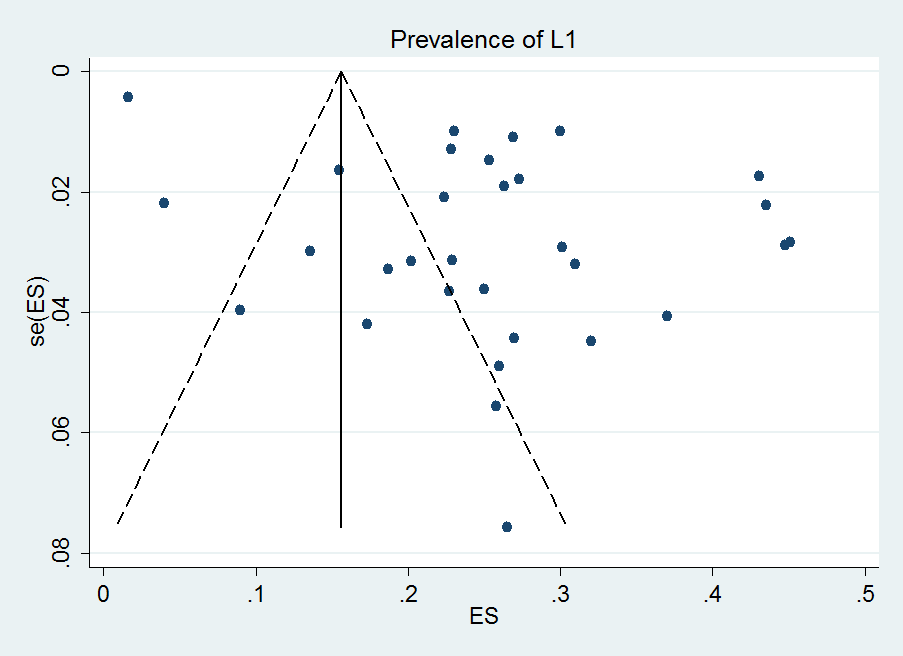


Prevalence of L2 *(forest plot and funnel plot for the whole population)*


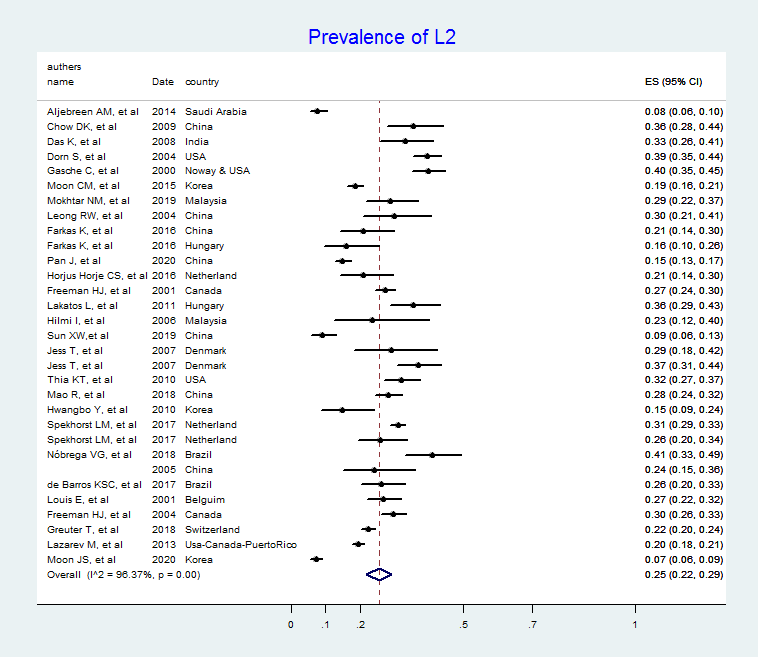


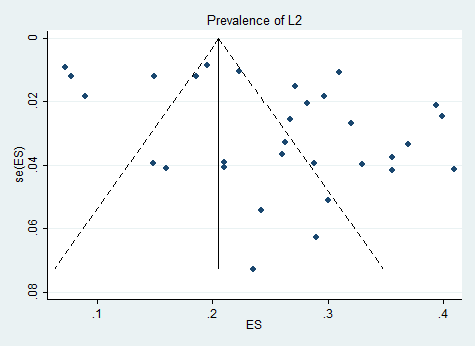


Prevalence of L3 *(forest plot and funnel plot for the whole population)*


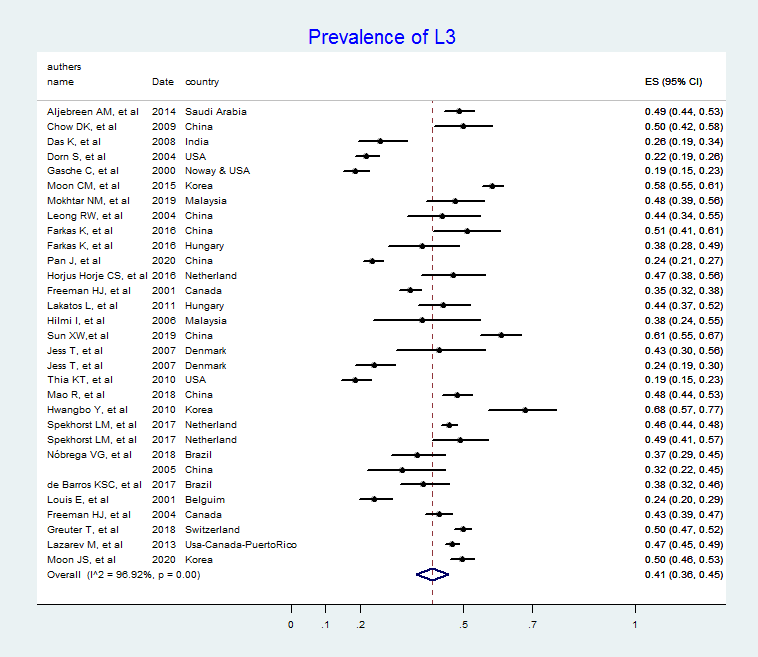


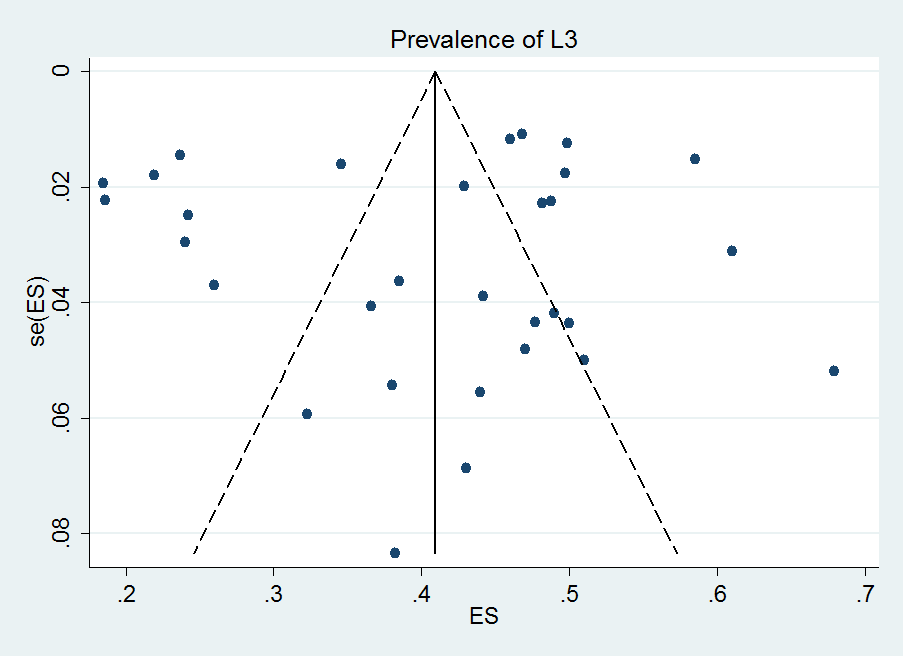


Prevalence of L4 *(forest plot and funnel plot for the whole population)*


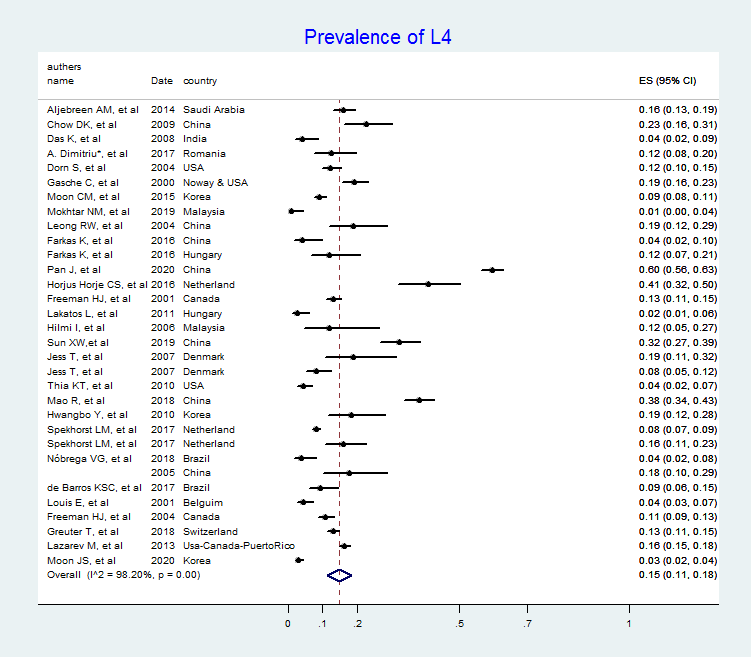


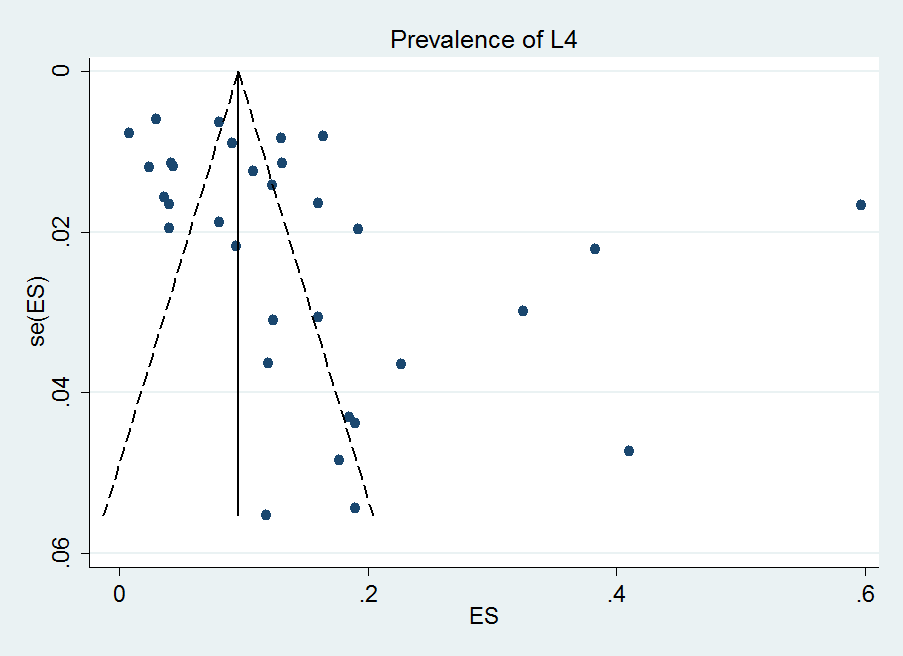


Prevalence of B1 *(forest plot and funnel plot for the whole population)*


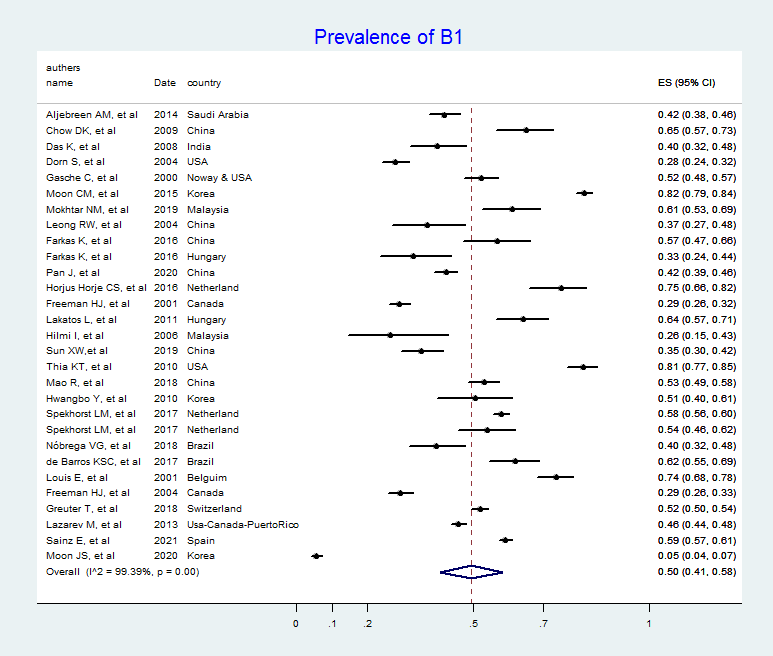


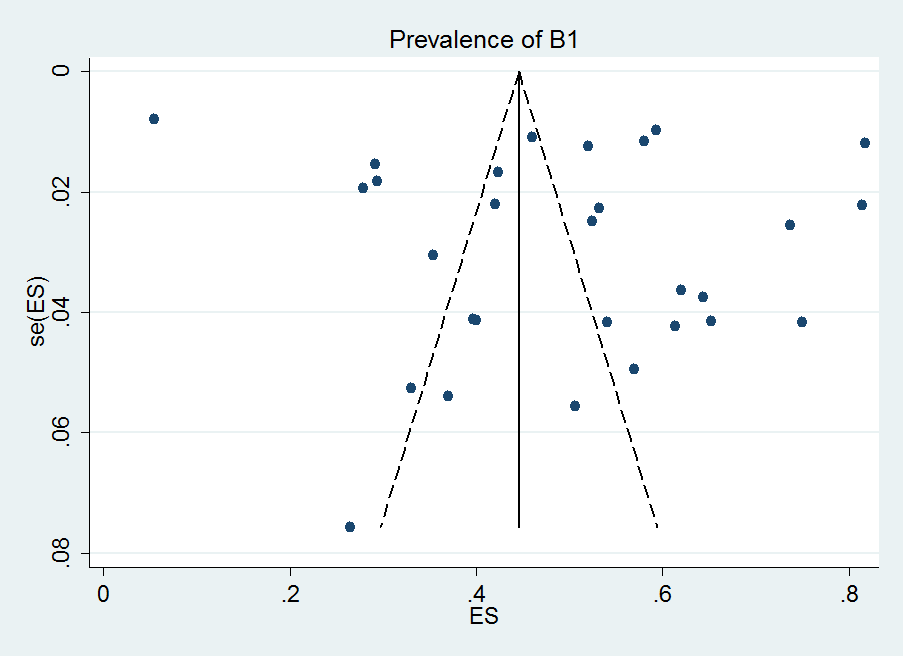


Prevalence of B2 *(forest plot and funnel plot for the whole population)*


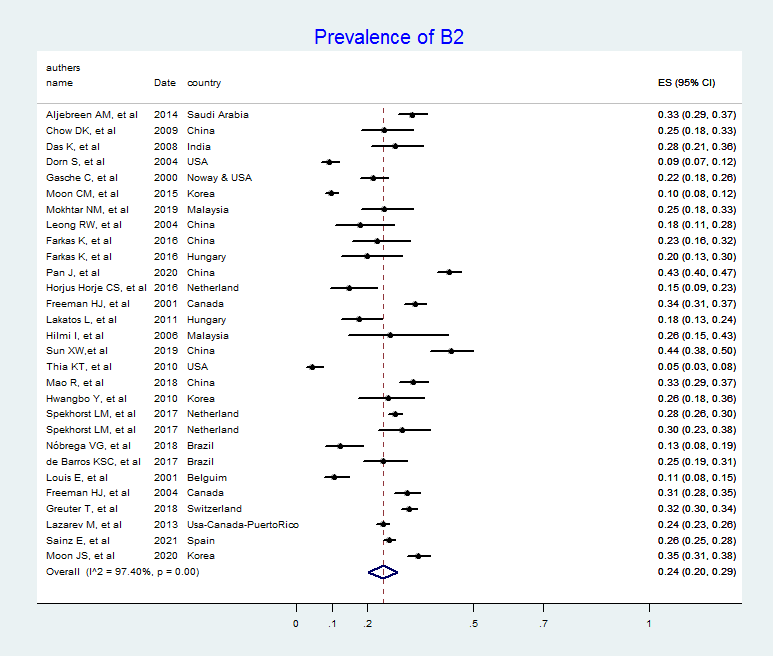


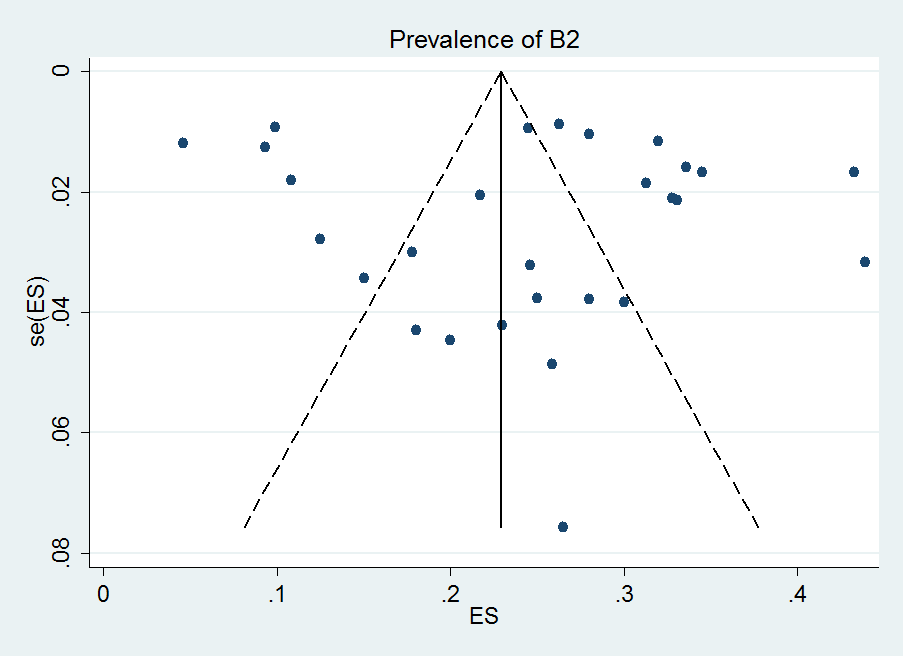


Prevalence of B3 *(forest plot and funnel plot for the whole population)*


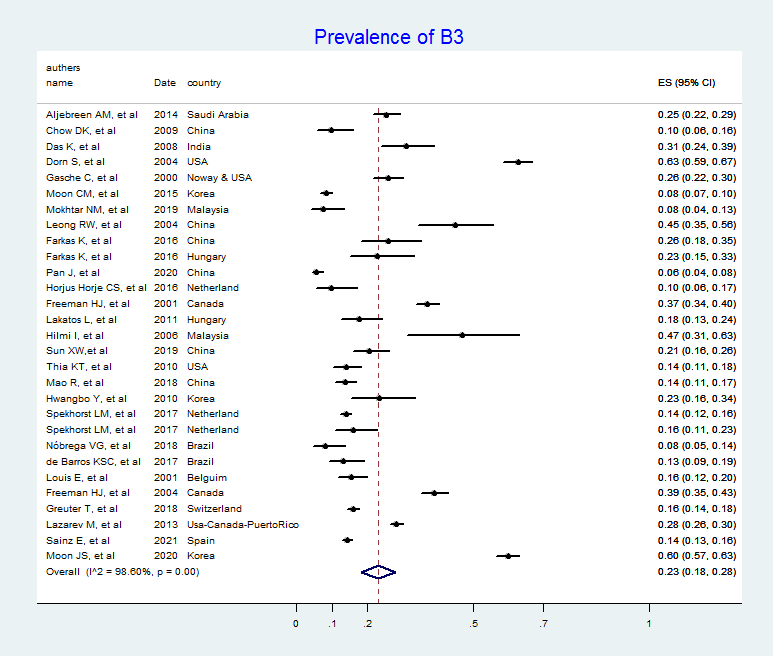


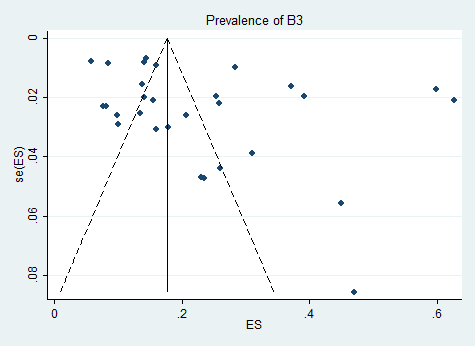


Prevalence of P (*forest plot and funnel plot for the whole population*)


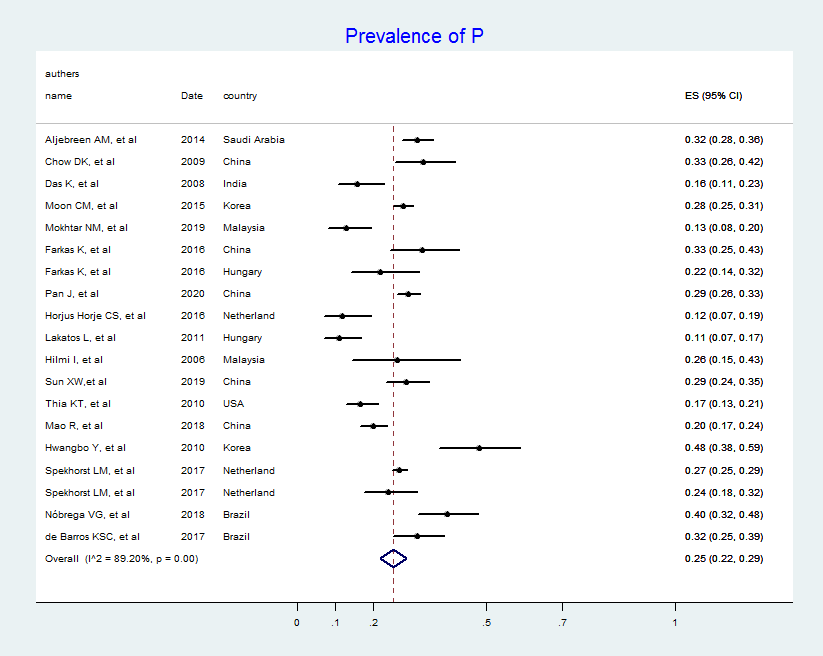


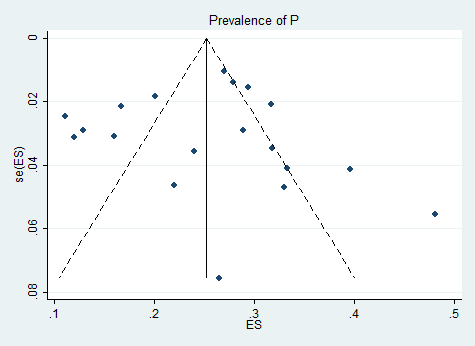


**All Forest Plots and Funnel Plots for prevalence Based on the L4 population (UGI-CD population)**

Prevalence of L1 *(forest plot and funnel plot in L4 Population)*


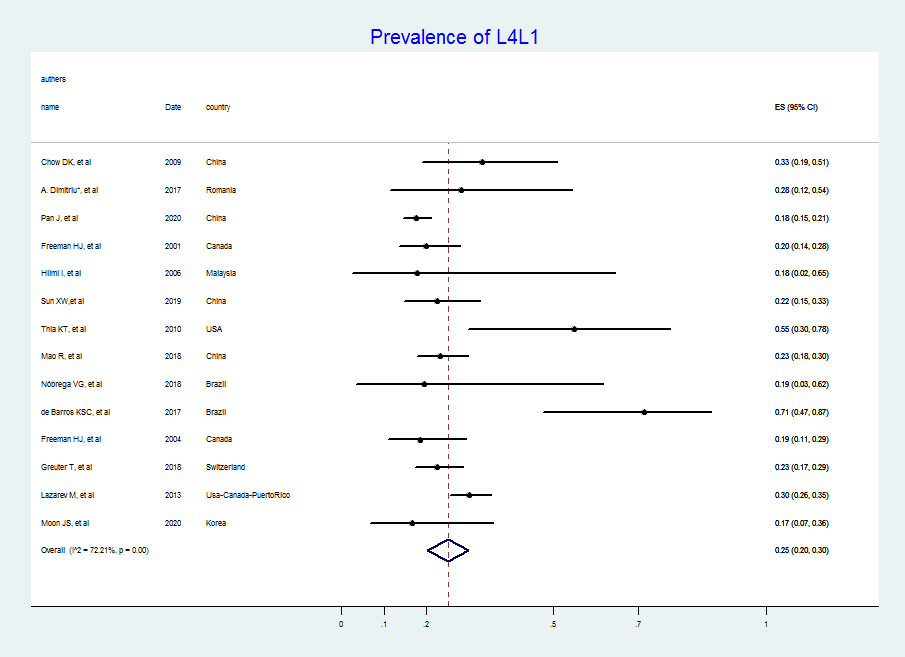


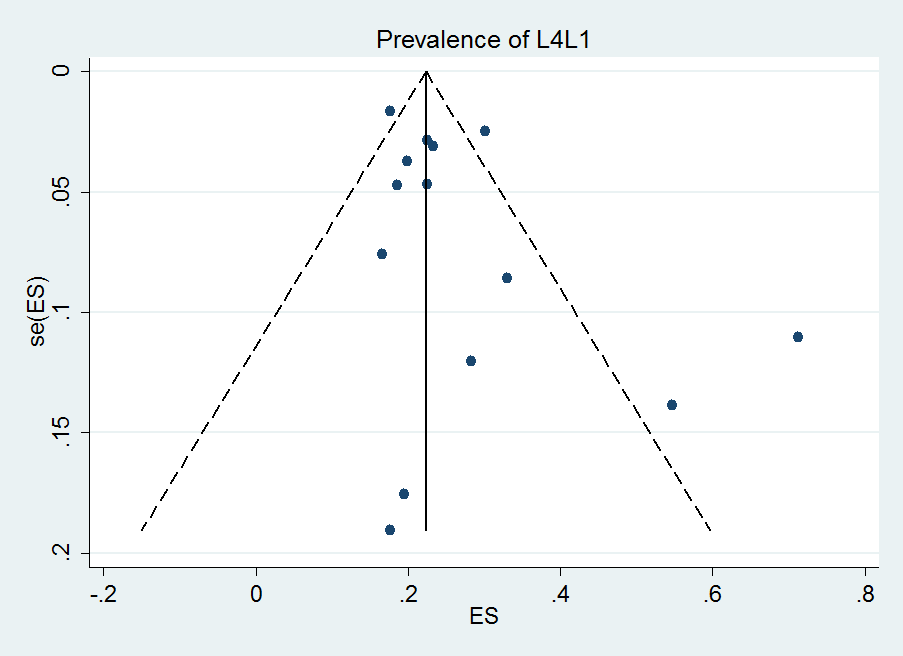


Prevalence of L2 *(forest plot and funnel plot in L4 Population)*


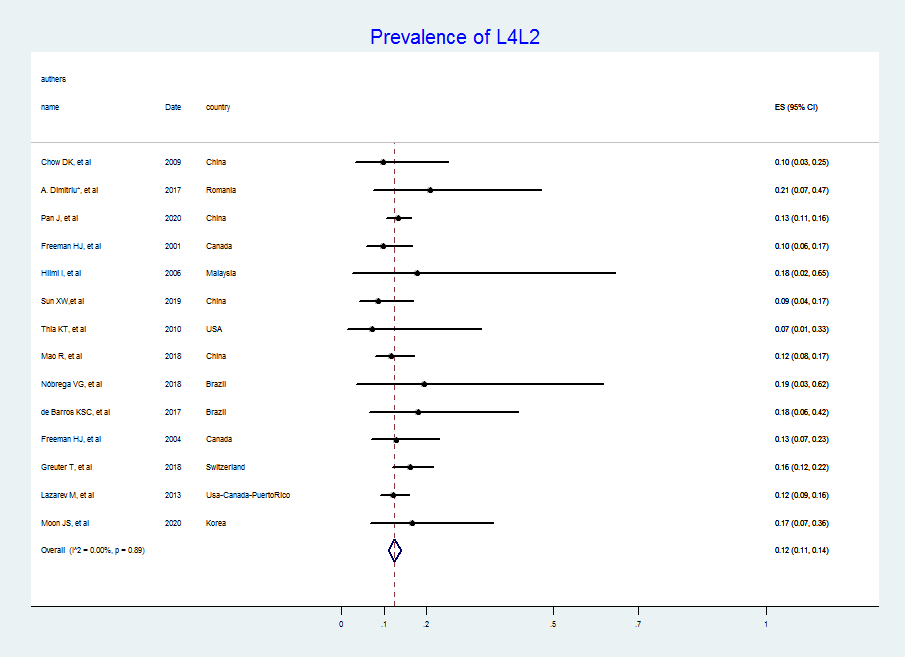


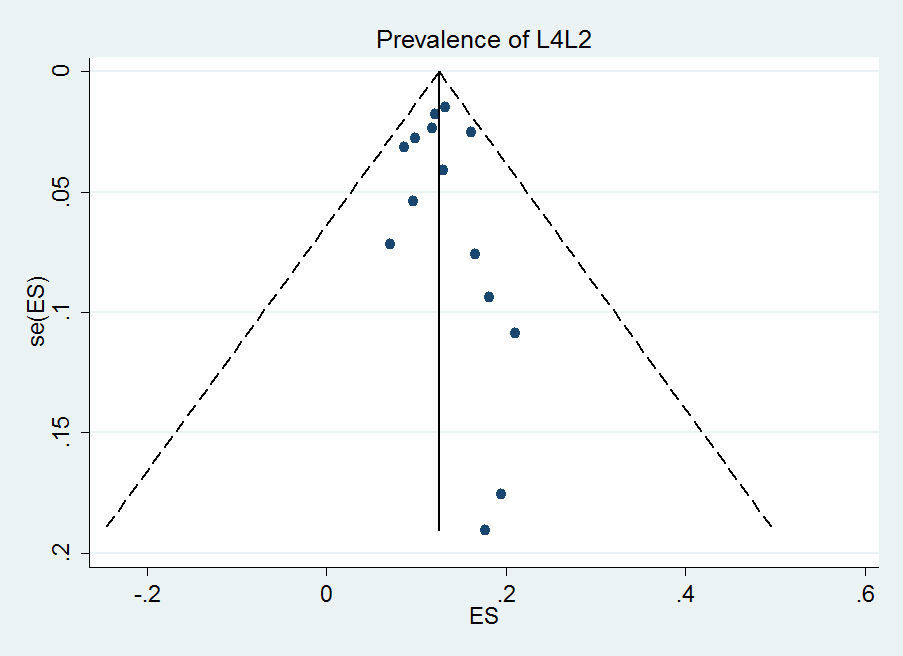


Prevalence of L3 (forest plot and funnel plot in L4 Population)


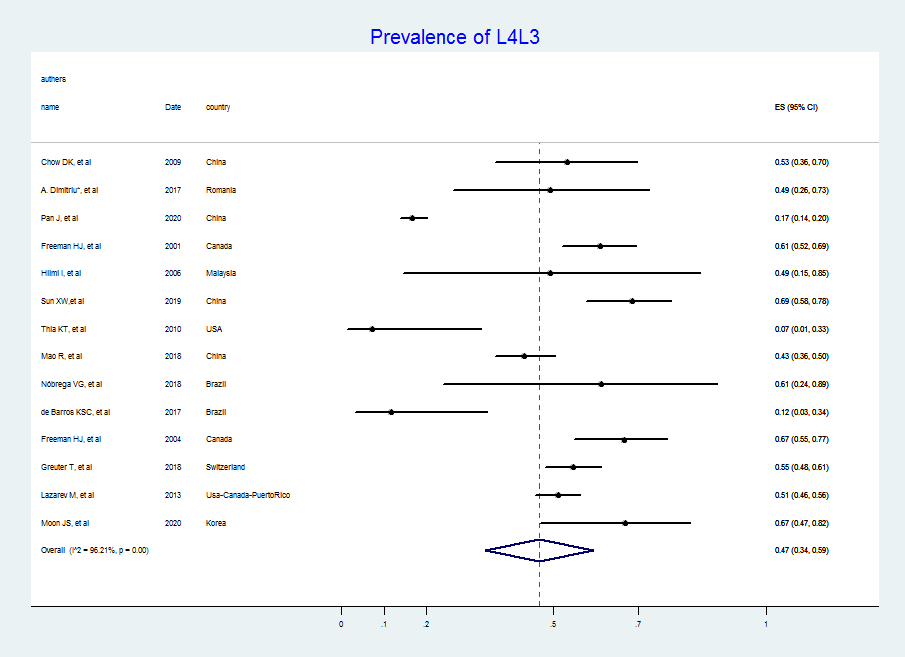


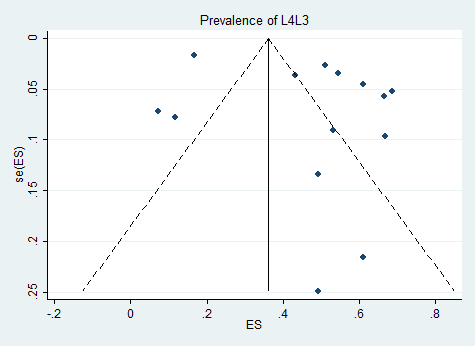


Prevalence of B1 (forest plot and funnel plot in L4 Population)


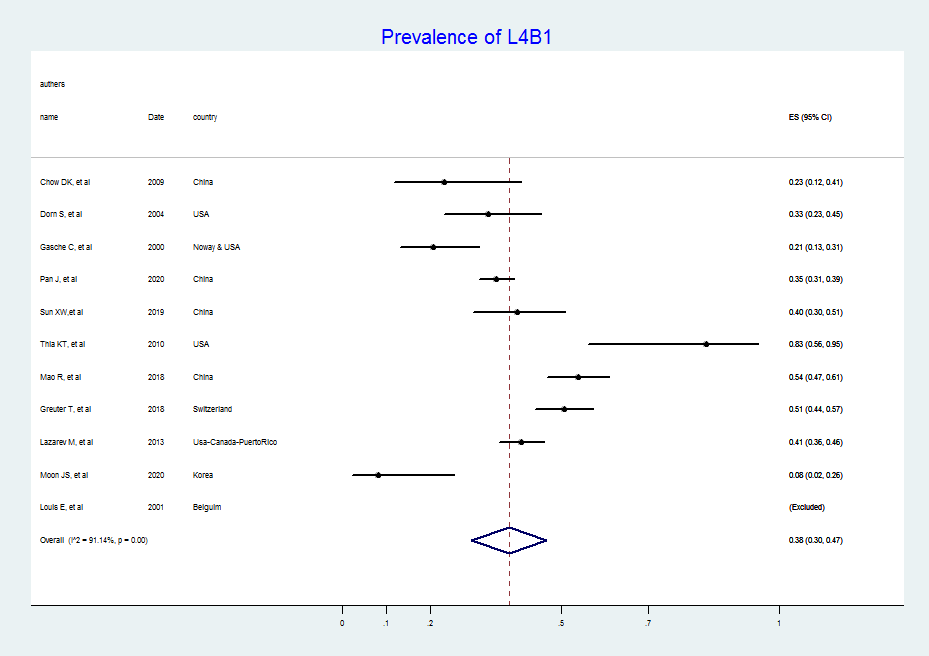


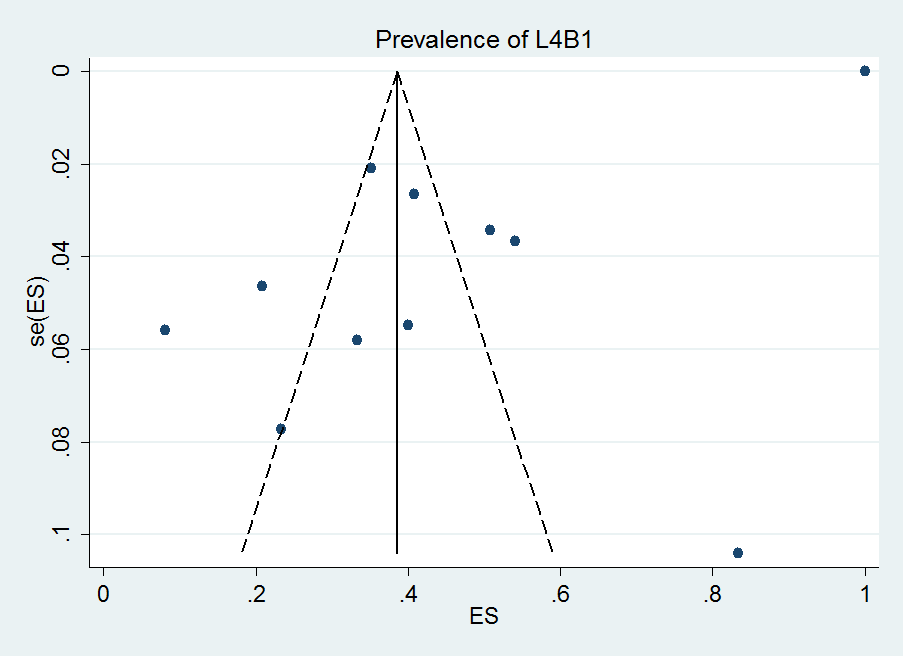


Prevalence of B2 (forest plot and funnel plot in L4 Population)


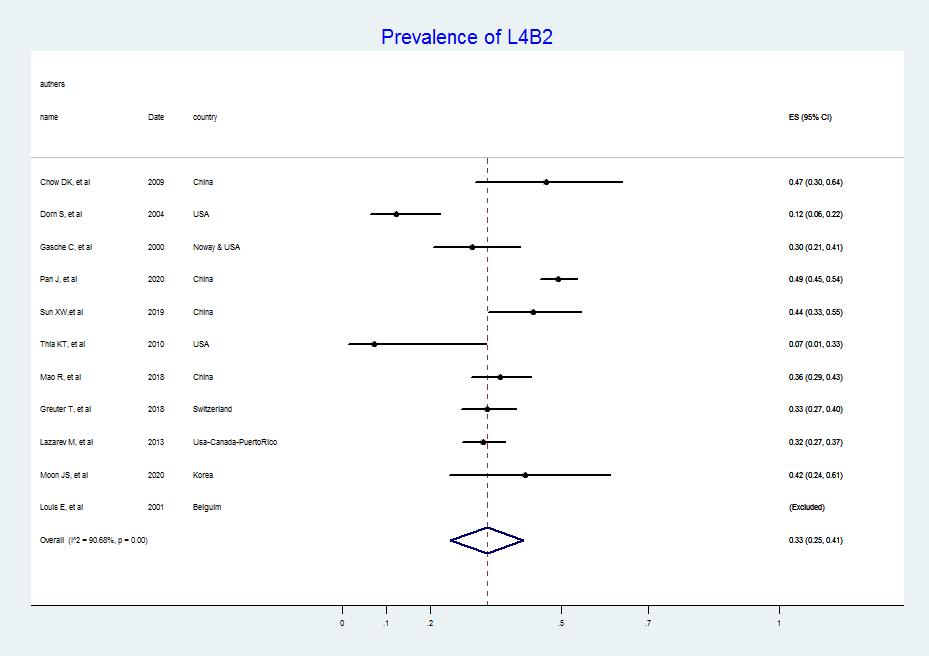


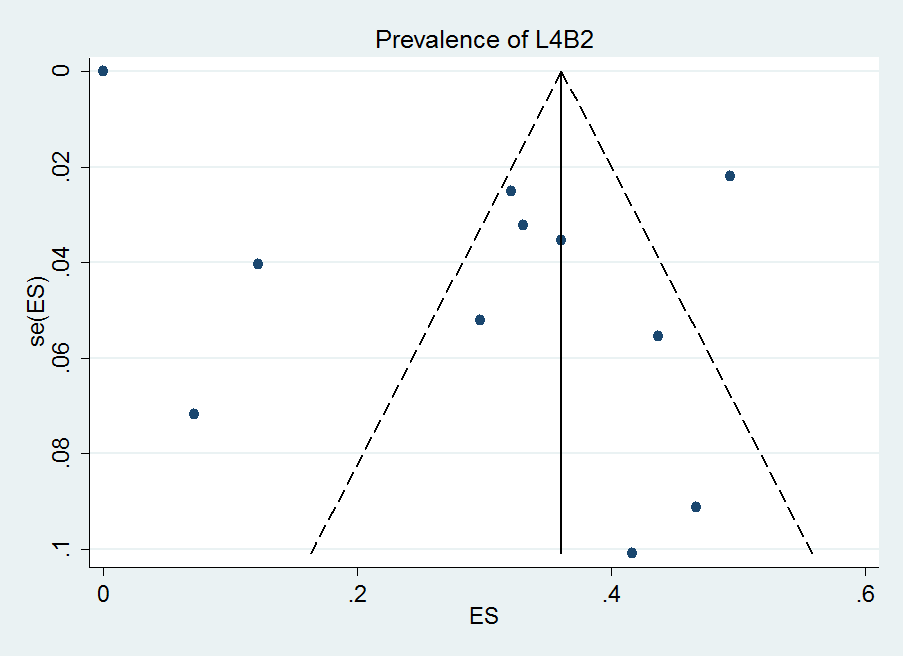


Prevalence of B3 (forest plot and funnel plot in L4 Population)


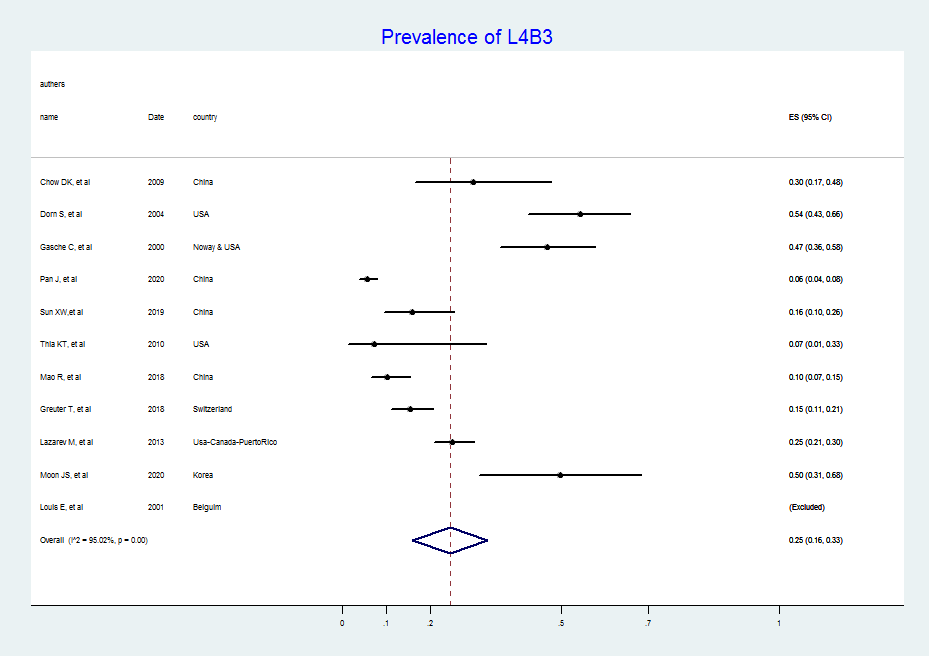


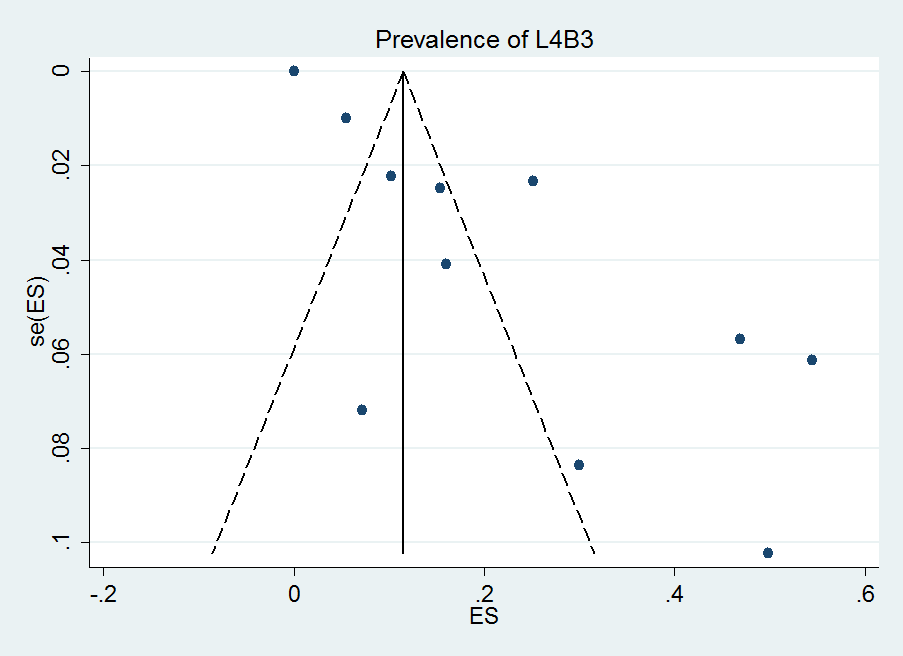


Prevalence of P (forest plot and funnel plot in L4 Population)


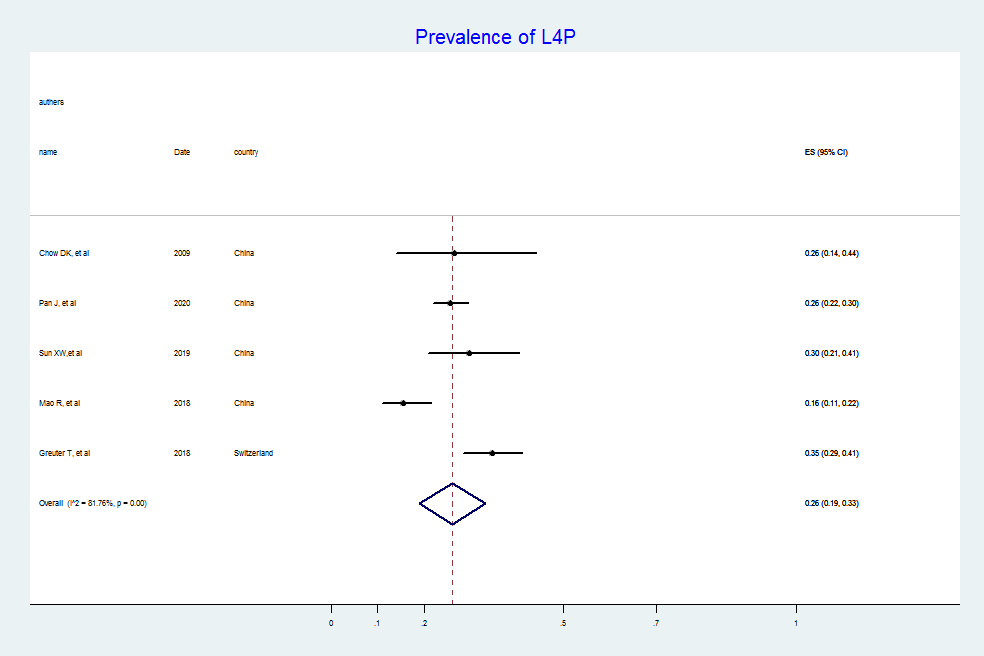


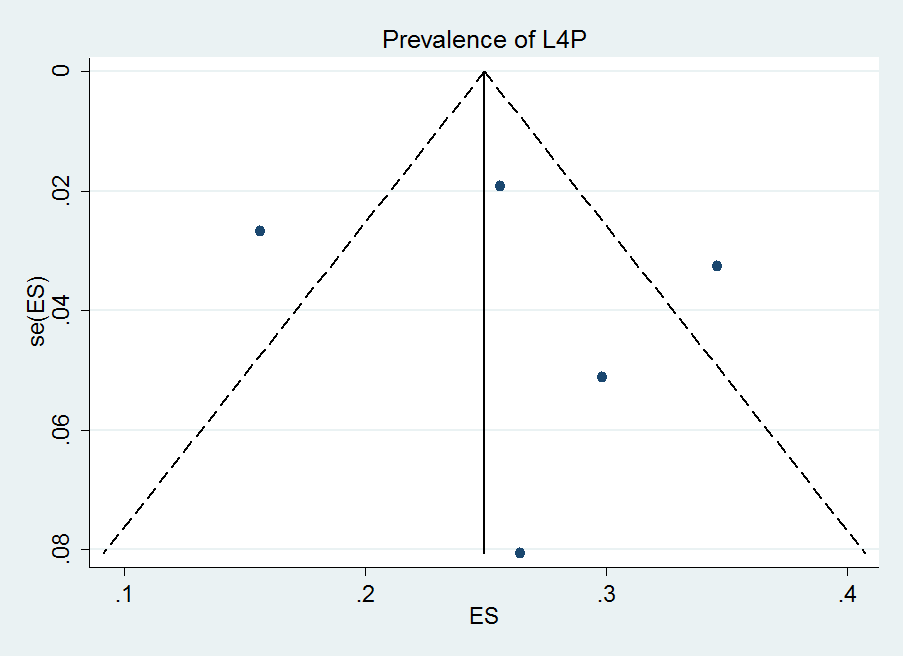


Prevalence of onlyL4 (forest plot and funnel plot in L4 Population)


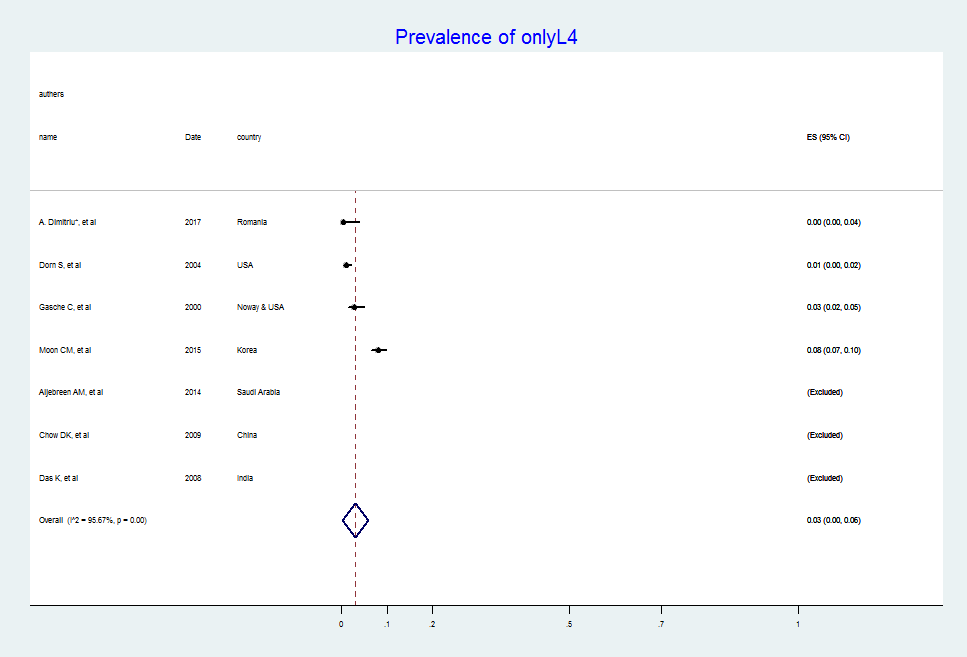


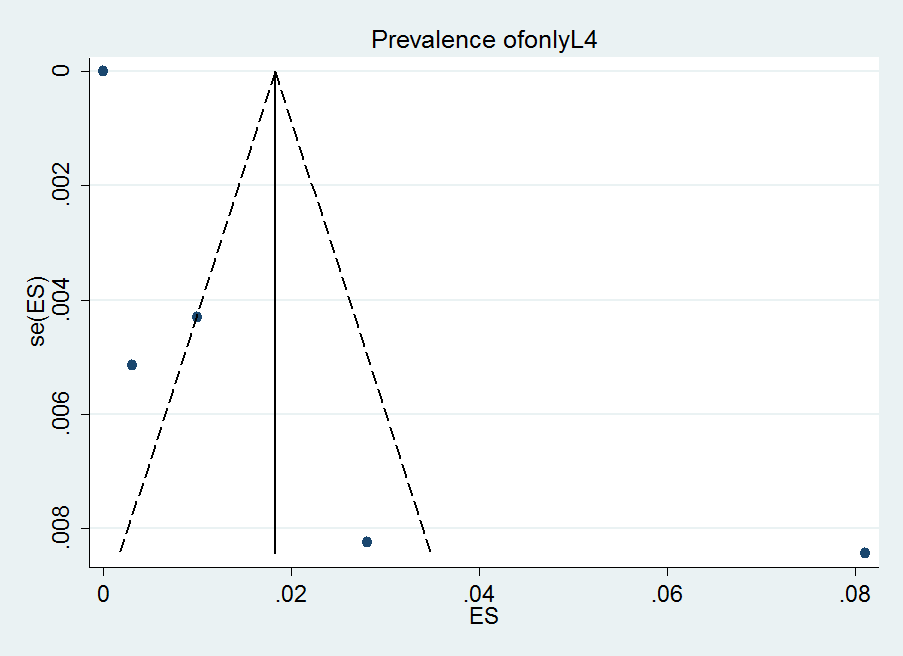


**Forest Plots for All Prevalence Based on Subgroups**

**1)** **forest plot for illustration of subgroup analysis based on the upper GI involvement – (L4 population vs non-L4 population)**

Prevalence of L1

Prevalence of L2

Prevalence of L3

Prevalence of B1

Prevalence of B2

Prevalence of B3

Prevalence of P

**2) Forest Plots for illustration of subgroup analysis based on region**

**(Asia vs Western)**

Prevalence of L1


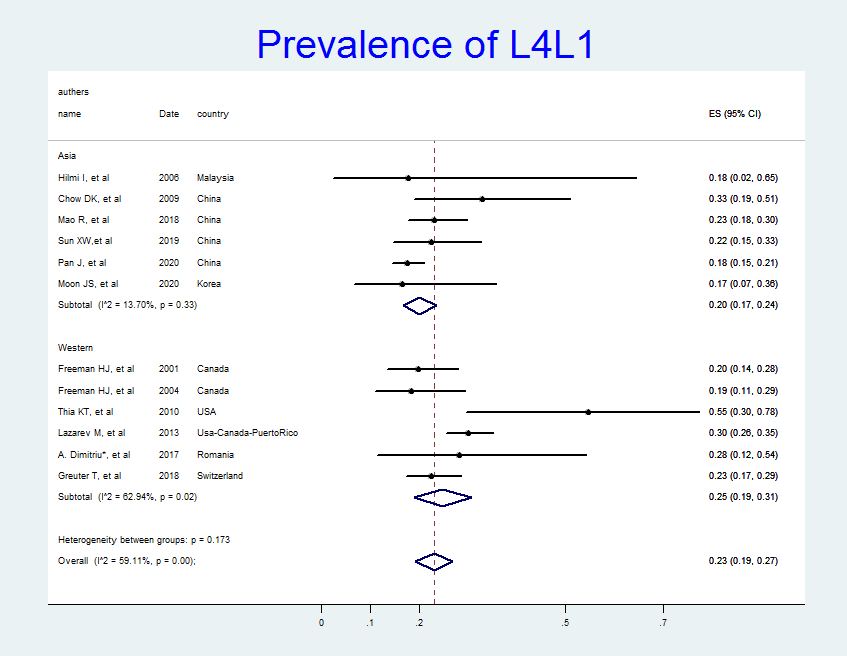


Prevalence of L2


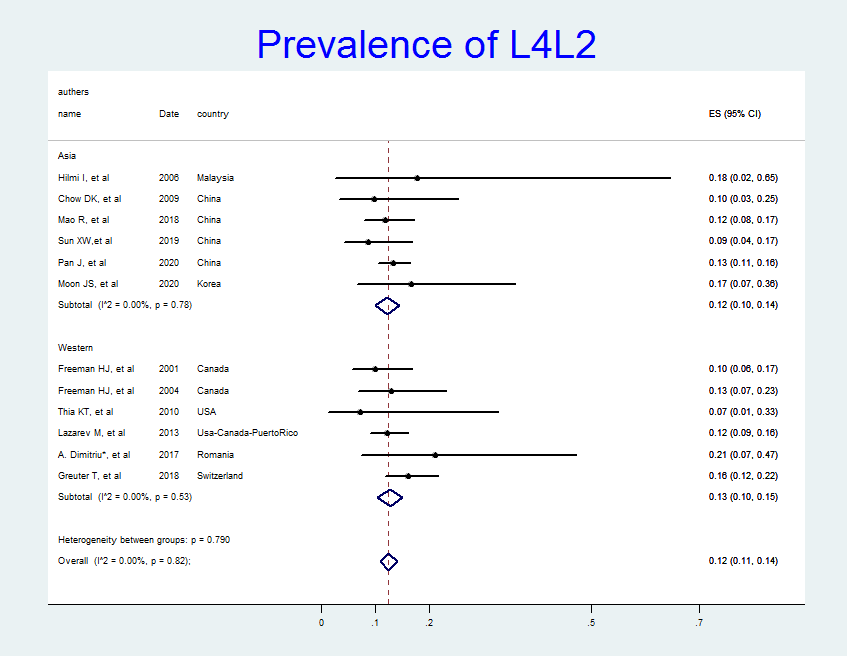


Prevalence of L3


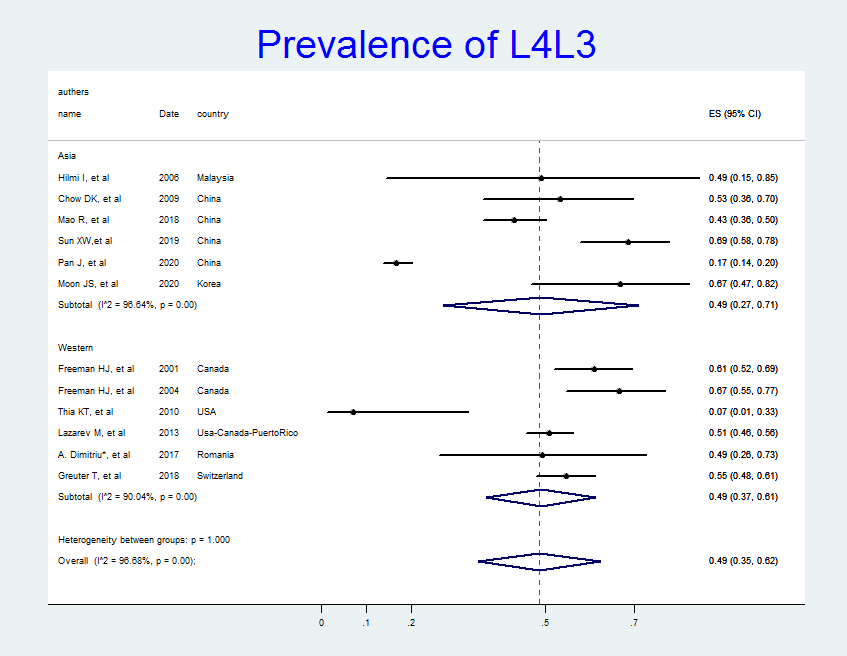


Prevalence of B1


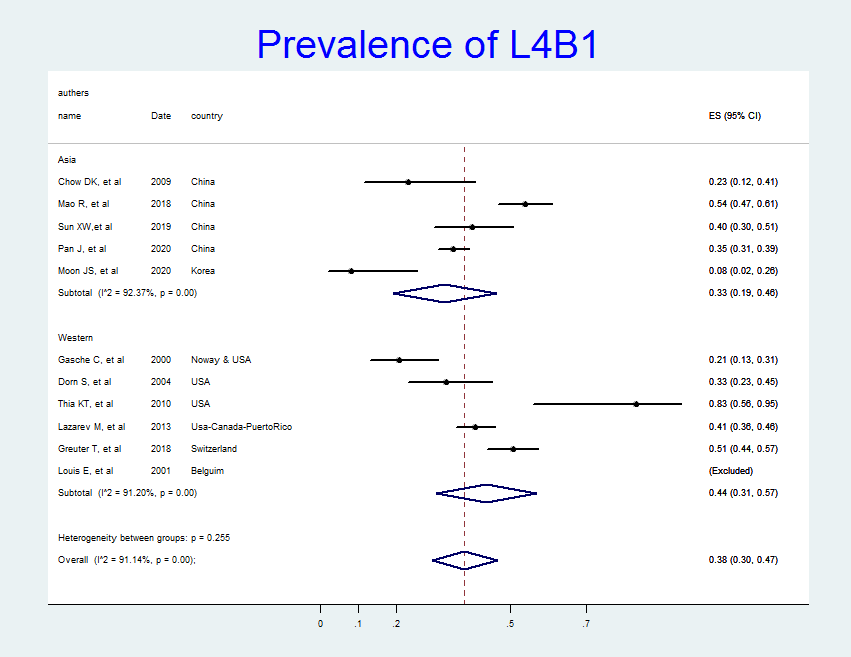


Prevalence of B2


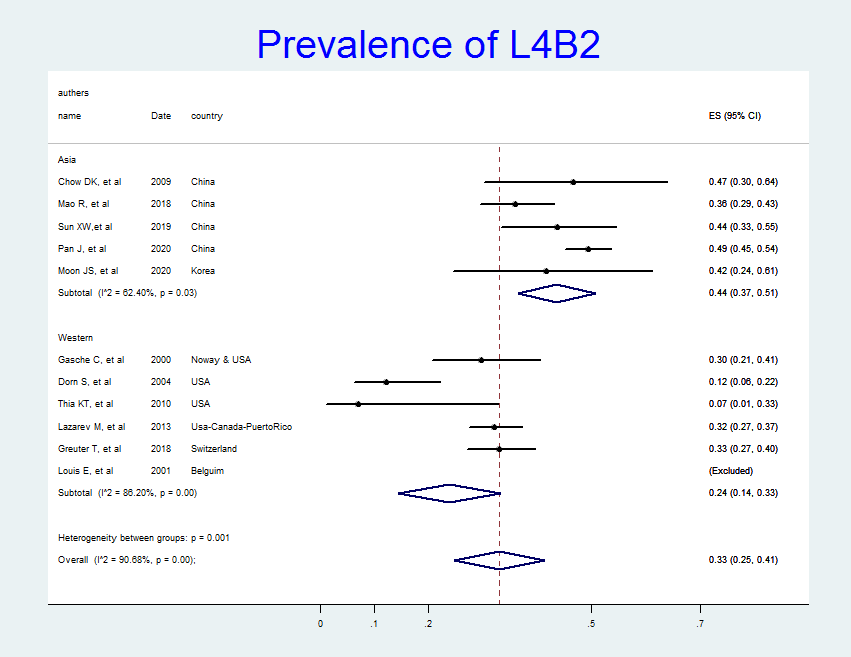


Prevalence of B3


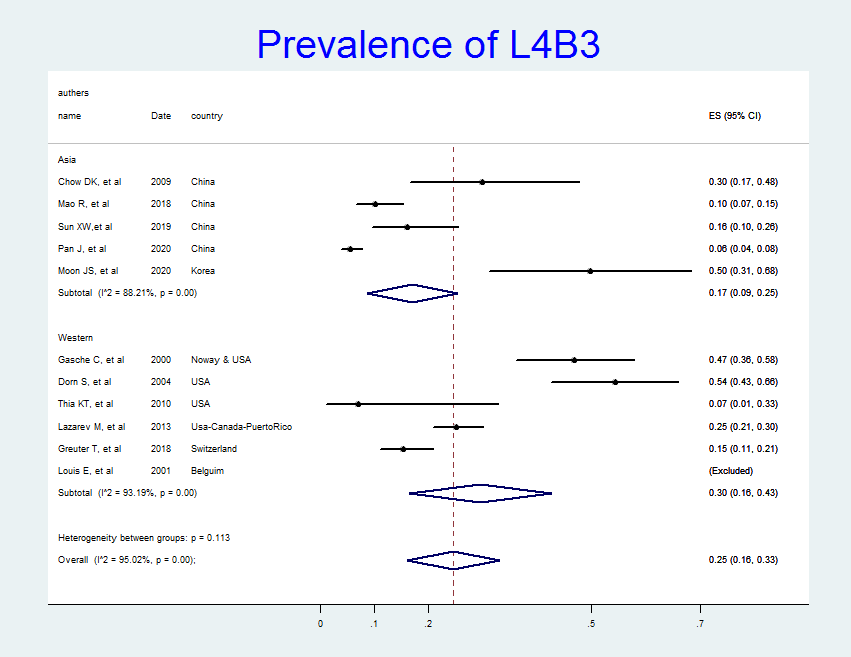


Prevalence of P


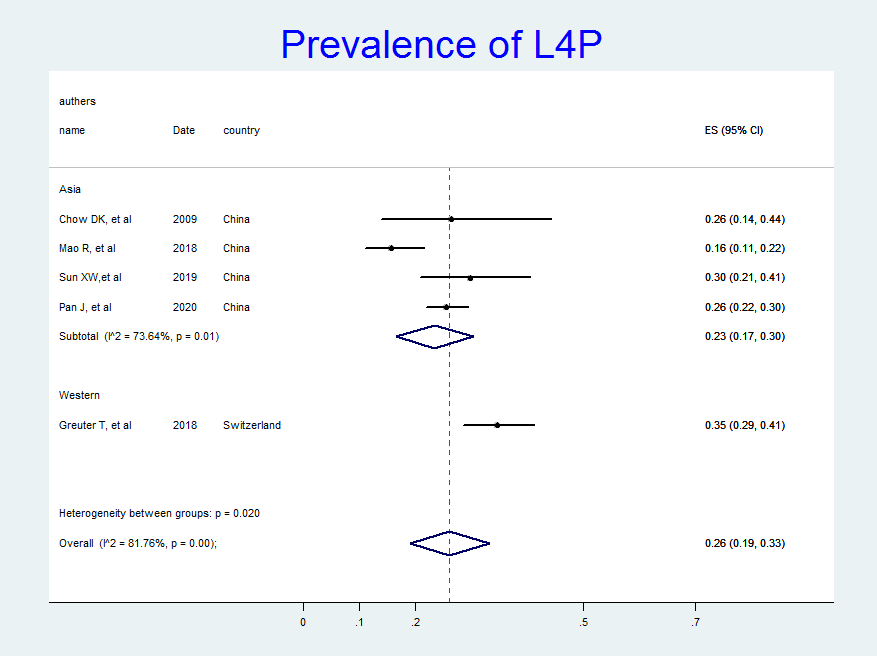

Supplement: Supplementary file 1 — Data S1. Supplementary file [file JGH3-7-325-s002.docx]
