# Supplementary material for: A systematic review and meta‐analysis of prevalence and clinical features of upper gastrointestinal (UGI) tract Crohn's disease in adults compared to non‐UGI types
Source: JGH Open. 2023 May 10;7(5):325–36. doi: 10.1002/jgh3.12888 (PMC10230113; doi:10.1002/jgh3.12888)
Supplement: Supplementary file 2 — Data S2. Supplementary file [file JGH3-7-325-s001.docx]

Our search lines were

1- ((upper gastrointestinal tract) OR (upper GI tract) OR (upper gastrointestinal tract)) AND ((Crohn’s disease) OR (Crohn disease) OR (Crohn* disease))

2- (UGI-CD) OR (UGI_CD)

In addition, we searched title and abstract of the papers in each database for MESH words presented in the search lines.
